# Enhanced Melanoma‐Targeted Therapy by “Fru‐Blocked” Phenyboronic Acid‐Modified Multiphase Antimetastatic Micellar Nanoparticles

**DOI:** 10.1002/advs.201800229

**Published:** 2018-07-13

**Authors:** Yang Long, Zhengze Lu, Ling Mei, Man Li, Kebai Ren, Xuhui Wang, Jiajing Tang, Zhirong Zhang, Qin He

**Affiliations:** ^1^ Key Laboratory of Drug Targeting and Drug Delivery Systems West China School of Pharmacy Sichuan University No. 17, Block 3, Southern Renmin Road Chengdu 610041 China

**Keywords:** antimetastatic nanoparticles, Fru‐blocking, LMWH, metastatic cascade, self‐delivering nanoparticles, d‐α‐tocopheryl succinate (TOS)

## Abstract

Metastasis remains the main driver of mortality in patients suffering from cancer because of the refractoriness resulting from the multi‐phase metastatic cascade. Herein, a multifunctional self‐delivering PBA‐LMWH‐TOS nanoparticle (PLT NP) is established that acts as both nanocarrier and anti‐metastatic agent with effects on most hematogenous metastases of cancers. The hydrophilic segment (low molecular weight heparin, LMWH) inhibits the interactions between tumor cells and platelets. The hydrophobic segment (d‐α‐tocopheryl succinate, TOS) could inhibit the expression of matrix metalloproteinase‐9 (MMP‐9) in B16F10 cells which is first reported in this article. Surprisingly, even the blank NPs showed excellent anti‐metastatic capacity in three mouse models by acting on different phases of the metastatic cascade. Moreover, the overexpression of sialic acid (SA) residues on tumor cells is implicated in the malignant and metastatic phenotypes of cancers. Thus, these 3‐aminophenylboronic acid (PBA)‐modified doxorubicin (DOX)‐loaded NPs offer an efficient approach for the treatment of both solid melanomas and metastases. Furthermore, a simple pH‐sensitive “Fructose (Fru)‐blocking” coping strategy is established to reduce the NP distribution in normal tissues and distinctly increases the accumulation in melanoma tumors. These micellar NPs consisting of biocompatible materials offer a promising approach for the clinical therapy of highly invasive solid tumors and metastases.

## 1 Introduction

Tumor metastasis, rather than the primary tumor itself, is the main cause of cancer‐related mortality,^1^, ^2^, ^3^, ^4^ and the shortage of antimetastatic drugs in clinics is a major obstacle to the treatment of invasive cancers. The development of a multifunctional drug delivery nanosystem that can both eliminate solid tumors in situ and suppress tumor metastasis could be greatly beneficial for the treatment of invasive tumors. However, it is very challenging to achieve such goals with one nanosystem. Existing strategies are mostly limited to combine therapy based on chemotherapy, gene therapy, and radiotherapeutics.^5^, ^6^, ^7^, ^8^ Moreover, the metastasis cascade is inherent to the generation process of most tumor metastases, especially lung metastases.^9^, ^10^, ^11^, ^12^ Therefore, an adjuvant‐therapy‐based strategy that effectively inhibits the metastasis cascade might greatly contribute to preventing the metastasis of most malignant tumors. The inhibition of only one phase of the metastatic cascade is inadequate to terminate metastasis as the cascade is a complicated multiphase process that is mediated by many factors, including tumor cell intravasation, survival in the lymphatic and vascular circulation, and extravasation and colonization of distant organs.^9^, ^10^, ^11^, ^12^ Therefore, multiphase‐targeted therapeutic strategies are beneficial for the inhibition of metastasis.

**Scheme 1:**
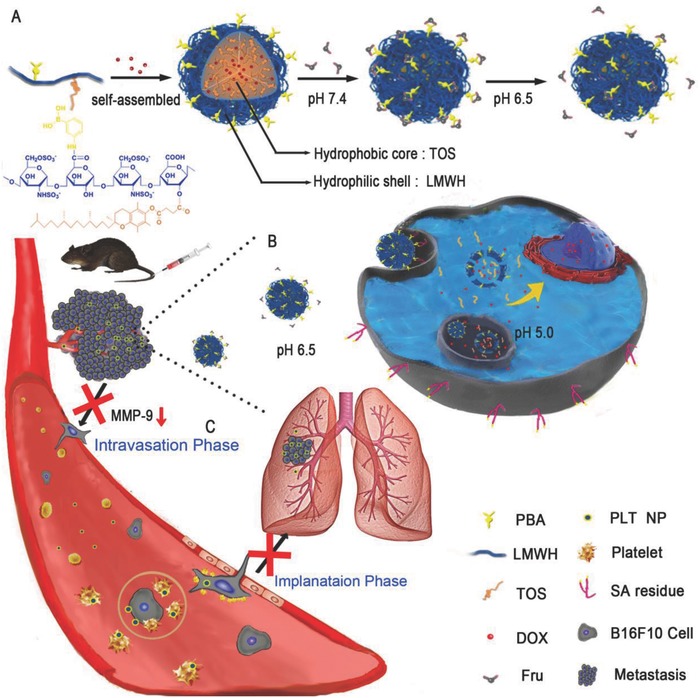
A) Schematic illustration of the preparation of PLT/ DOX NPs and the “Fru‐blocking” strategy. B,C) Schematic illustration of PLT/DOX NPs targeting a melanoma solid tumor and inhibiting lung metastasis.

Establishing multifunctional nanoparticles (NPs) to act as both nanocarriers and antimetastatic agents that affect most hematogenous metastases of cancers might be a promising strategy. Abundant experimental evidence has indicated that platelets support tumor metastasis by adhering to tumor cells to guard the cells from flow shear stress and immune elimination in blood vessels, helping the cells cross the blood vessel endothelium.^12^, ^13^, ^14^ Platelet secretions also assist tumor cell survival and proliferation within specific distant organs.^15^, ^16^ Remarkably, a few clinical trials have demonstrated the favorable effects of low‐molecular‐weight heparin (LMWH) on human tumors.^17^ LMWH inhibits the adhesion between platelets and tumor cells by hindering P‐selectin on activated platelets,^17^, ^18^, ^19^, ^20^ thus inhibiting the final stage of the metastatic cascade. Moreover, d‐α‐tocopheryl succinate (TOS), the most effective form of vitamin E analogues, can inhibit the drug resistance of tumors and increase the apoptosis of various types of cancer cells,^21^, ^22^, ^23^ including B16F10 melanoma cells.^24^ In this study, α‐TOS was confirmed to inhibit the expression of matrix metalloproteinase‐9 (MMP‐9) in B16F10 cells significantly. MMP‐9, an enzyme secreted by various tumor cells,^1^, ^25^, ^26^ including B16F10 cells,^27^, ^28^, ^29^ can degrade the extracellular matrix, facilitate detachment and invasion, and enhance tumor cells entry into blood vessels. Thus, TOS can inhibit the beginning of the metastatic cascade, which is a crucial stage of metastasis.^30^ Based on the above, we constructed a simple self‐delivering micellar NP consisting of the antimetastatic components LMWH and TOS, which inhibit different phases of the metastatic cascade (**Scheme** **1**C). This self‐delivering NP exhibited excellent antimetastatic effects in B16F10, 4T1, and CT26 metastatic mouse models without being loaded with any other antimetastatic agents or redundant components.

**Figure 1:**
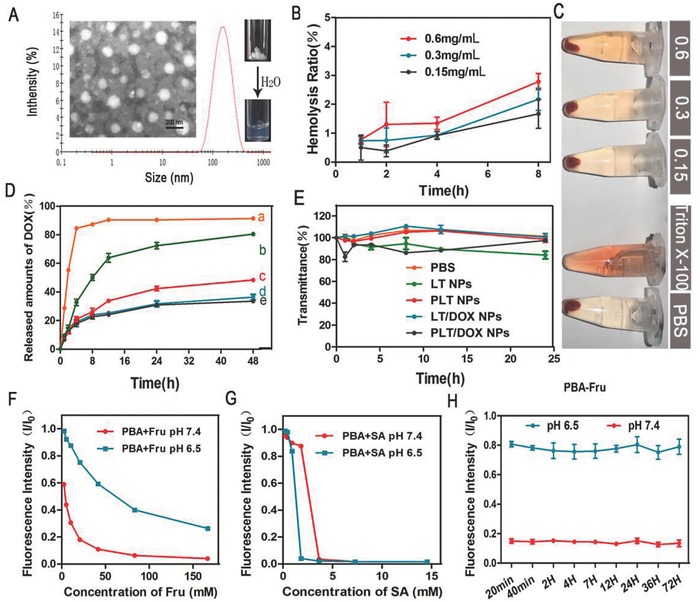
Characterization of NPs and investigation of the combination between Fru, SA, and PBA. A) Dynamic light scattering (DLS) size distribution and transmission electron microscopy (TEM) image of PLT NPs. B) Hemolysis ratio (%) and C) photo images of red blood cells cultured with PLT NPs at various concentrations. A hemolysis ratio less than 5% was regarded as no obvious hemolysis. (Means ± SD, *n* = 3). D) Cumulative DOX release of LT/DOX NPs and PLT/DOX NPs in phosphate buffered saline (PBS) at 37 °C. ((a) Free DOX in pH 7.4 PBS, (b) PLT/DOX NPs in pH 5.0 PBS, (c) PLT/DOX NPs in pH 6.5 PBS, (d) PLT/DOX NPs in pH 7.4 PBS, (e) LT/DOX NPs in pH 7.4 PBS) (means ± SD, *n* = 3). E) The serum stability of NPs during 24 h incubation with 50% FBS at 37 °C (means ± SD, *n* = 3). F) The affinity of free PBA to Fru and G) to free SA in pH 7.4 and pH 6.5 PBS and H) the stability testing of PBA‐Fru conjugate at different time points. The concentration of Fru was 20 × 10^−3^ m and the concentration of PBA was 60 × 10^−6^ m.

In addition, sialylation has long been associated with metastatic cell behaviors, including invasion and immune evasion.^31^, ^32^, ^33^, ^34^ The overexpression of sialic acid (SA) residues on cell membranes has been implicated in the malignant and metastatic phenotypes of various types of cancers.^35^ To optimize the antimetastatic self‐delivering micellar NPs, 3‐aminophenylboronic acid (PBA) was conjugated to LMWH to form PBA‐LMWH‐TOS nanoparticles (PLT NPs), endowing the nonselective micelles with high metastatic tumor cell‐targeting capacity.^36^, ^37^, ^38^ The small molecule PBA binds to SA residues with high affinity and has certain advantages, such as low cost, high stability, low cytotoxicity, and ease of modification. Unfortunately, some normal tissues, especially liver and lung tissues, also express SA residues to some degree, which inevitably causes off‐target effects.^39^ There are many differences between normal tissues and tumor microenvironments, such as the high expression of many proteases, the high interstitial pressure, incomplete capillary vessels, the low oxygen content, and the low pH of tumor microenvironments. Utilizing these differences to design a tumor‐microenvironment‐exposed targeting ligand might contribute to the improvement of targeting efficiency. Inspired by the use of a PBA‐modified nanostructure to reversibly capture and release tumor cells in vitro,^40^, ^41^ we established a very simple strategy to reduce the off‐target effects of NPs. PBA can form reversible, pH‐sensitive borate esters with *cis*‐diol‐containing monosaccharides (e.g., glucose, galactose, and d‐fructose),^42^, ^43^ but formed stable links with SA residues, even under intratumoral pH conditions, that is, pH 6.5 (**Figure** **1**G).^36^ Therefore, we hypothesized that coincubating PLT NPs with monosaccharides beforehand to block PBA could maintain the stability at a physiological pH of 7.4 , inhibiting the interaction between PBA and SA residues in normal tissues. Upon arriving in the acidic environment of the tumor site, PBA could be re‐exposed and could regain its SA‐targeting capacity specifically at the tumor site (Scheme 1A,B). Doxorubicin (DOX) is a typical chemotherapeutic drug with severe side effects, especially cardiotoxicity. Hence, we chose DOX as a model drug to investigate the attenuation of toxicity by NPs and the “Fru‐blocking” strategy for off‐target side effects.

## 2 Results and Discussion

### 2.1 Construction of LMWH‐TOS and PBA‐LMWH‐TOS Conjugates

To construct the self‐delivering micellar NPs, we designed two amphiphilic conjugates, LMWH‐TOS and PBA‐LMWH‐TOS. Carboxyl groups and hydroxyl groups were employed skillfully in the designing of the structures. First, LMWH‐TOS was successfully synthesized by conjugating TOS (—COOH) to LMWH (—OH) via the formation of an ester bond (Figure S1A, Supporting Information). The product was dissolved in D_2_O and DMSO‐d6 to confirm the hydrophilic and hydrophobic segments, respectively. The characteristic peaks of LMWH (3.2–5.5 ppm, Figure S2B, Supporting Information) and TOS (1.0–3.0 ppm, Figure S2D, Supporting Information) in the ^1^H‐NMR spectrum of the product indicated the successful conjugation of TOS to LMWH (Figure S2E,F, Supporting Information). The LMWH content in the LMWH‐TOS conjugate was then quantified chemically using toluidine blue spectrophotometry (Figure S4A, Supporting Information). The LMWH content in the LMWH‐TOS conjugate was 28.1% (w/w).

In addition, the PBA‐LMWH conjugate was synthesized by conjugating PBA to LMWH via an amidation reaction between the primary amine group of PBA and the carboxylic acid groups of LMWH (Figure S1B, Supporting Information). The characteristic peaks of aromatic protons ((d,e) 7.3–7.4 ppm; (c,f) 7.6–7.7 ppm) in the ^1^H‐NMR spectrum of the final product indicated the successful synthesis of the PBA‐LMWH conjugate (Figure S2C, Supporting Information). PBA‐LMWH‐TOS was synthesized by conjugating TOS to PBA‐LMWH via an ester bond as described above. The successful synthesis of PBA‐LMWH‐TOS conjugate was confirmed by MS and IR. The characteristic peaks of PBA ([M+H]^+^ = 138.1) and TOS ([M+H]^+^ = 531.5, [M+NH_4_]^+^ = 548.5) were identified in the mass spectrum of PBA‐LMWH‐TOS conjugate (Figure S3A–C, Supporting Information). Moreover, in the IR spectrum of PBA‐LMWH‐TOS conjugate, obvious characteristic absorption peaks of TOS and PBA were observed (Figure S3D–G, Supporting Information). Due to the fluorophore present, PBA could be detected at an excitation wavelength of 302 nm and an emission wavelength of 375 nm. To calculate the PBA content, a simple linear regression method (Figure S4B, Supporting Information) was established using a fluorospectrophotometer. The PBA content in the PBA‐LMWH‐TOS conjugate was 2.13% (w/w).

### 2.2 Preparation and Characterization of DOX‐Loaded NPs

In PBS, the amphiphilic LMWH‐TOS and PBA‐LMWH‐TOS conjugates self‐assembled into well‐defined NPs with hydrophilic surfaces and hydrophobic cores. Dynamic light scattering (DLS) detection and transmission electron microscopy (TEM) imaging (Figure 1A and Figure S5, Supporting Information) revealed spherical NPs with diameters of ≈140 nm, which enhanced the superior tumor accumulation capacity of the NPs owing to the enhanced permeability and retention (EPR) effect. A series of ratios of LMWH to TOS were investigated to obtain a suitable particle size (Table S1, Supporting Information). The experimental results showed that when the total number of carboxyl groups of TOS was equal to the total number of hydroxyl groups of LMWH, the NPs had a homogeneous mean diameter of 140 nm with a narrow size distribution (PDI = 0.142). The DOX‐loading capacity and encapsulation efficiency were 8–9% and 88–91%, respectively (Table S2, Supporting Information). The introduction of PBA did not greatly change the zeta potential. The LMWH‐TOS NPs (LT NPs) and PLT NPs were electronegative (Table S1, Supporting Information). Moreover, blood erythrocytes, as the most abundant cells in blood, play an essential role as oxygen carriers in systemic circulation. However, heparin has commonly been used as an anticoagulant and may cause NPs to induce hemolysis as a side effect. Thus, a hemolysis assay was conducted. The result showed that NPs had only a slight influence on blood erythrocytes, and the hemolysis ratio was consistently lower than 5% within the concentration range of 150*–*600 µg mL^−1^ (Figure 1B,C and Figure S6, Supporting Information). The interactions between NPs and blood components can change the particle size. However, the serum stability assay showed that the LT NPs and PLT NPs remained stable in 10 and 50% FBS (Figure 1E and Figure S7, Supporting Information), and the transmittance showed no detectable change within 24 h.

### 2.3 In Vitro Drug Release

In order to investigate the drug release behavior of LT/DOX NPs and PLT/DOX NPs, an in vitro drug release assay was conducted. As shown in Figure 1D, both LT/DOX NPs and PLT/DOX NPs did not significantly release DOX in pH 7.4 PBS, which indicated that these NPs could remain stable under physiological conditions. After 48 h, PLT/DOX NPs released about 40% DOX under pH 6.5 conditions, and this ratio increased to about 80% under pH 5.0 conditions. This result suggested that the drug release behavior of PLT/DOX NPs was acid‐dependent, and this feature may be due to the ester bond groups in PLT conjugate. Therefore, PLT/DOX NPs could release DOX rapidly under lysosomal internal environment (about pH 5.0).

### 2.4 Extracellular pH‐Activated Cellular Uptake Analysis of the NPs In Vitro

First, the binding characteristics of Fru and SA with PBA in vitro were investigated. PBA contains a fluorophore that exhibits decreased fluorescence when PBA binds with sugars or SA.^36^ Thus, the affinity of Fru and SA to PBA could be evaluated by the variation in fluorescence intensity. According to the affinity assay (Figure 1F,G), the affinity of Fru to PBA at pH 7.4 was stronger than the affinity at pH 6.5. However, the binding of PBA and SA was not influenced by the pH. The binding stability assay showed rapid binding and relatively stable affinity between PBA and Fru at pH 7.4 (Figure 1H).

**Figure 2:**
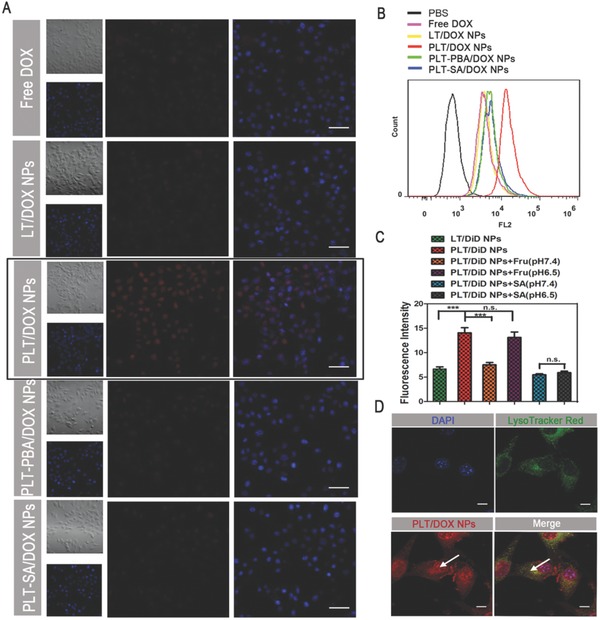
Cellular uptake of B16F10 cells after incubation with different preparations. A) Confocal laser scanning microscopy (CLSM) images of B16F10 cells after incubation with free DOX, LT/DOX NPs, PLT/DOX NPs, PLT‐BA/DOX NPs, or PLT‐SA/DOX NPs for 2 h. The competitive inhibition assay was performed by preincubating with free PBA or free SA for 1 h and observing the DOX channel (red) and DAPI‐stained nucleus channel (blue). The scale bar represents 100 µm. B) The representative histograms of competitive uptake assay analyzed by flow cytometry (FACS). C) Quantitative cellular uptake of B16F10 cells after incubation with LT/DiD NPs, PLT/DiD NPs, PLT‐Fru/DiD NPs, and PLT‐SA/DiD NPs for 2 h at pH 6.5 and pH 7.4, respectively (means ± SD, *n* = 3, *** indicates *p* < 0.001). D) CLSM images of B16F10 cells after incubation with PLT/DOX NPs for 2 h, showing LysoTracker‐stained lysosome channel (green), DOX channel (red), and DAPI‐stained nucleus channel (blue). The arrow in the left indicated the signal of DOX in nucleus and the arrow in the right indicated the colocalization of DOX and lysosome. The scale bar represents 10 µm.

The comparison of cellular accumulation efficiency between LT NPs and PLT NPs was evaluated on B16F10 and HepG2 cells overexpressing SA residues and COS‐7 cells with low expression of SA residues.^40^ The cellular uptake of DOX from PLT/DOX NPs by B16F10 (**Figure** **2**A) and HepG2 cells was significantly higher than that from LT/DOX NPs (*p* < 0.01) (Figures S8 and S9, Supporting Information), while there was no significant difference observed with COS‐7 cells (Figures S10 and S11, Supporting Information). As all NPs exhibited similar particle sizes and zeta potentials (Figure S5, Supporting Information), the results suggested that PBA, which has a high affinity for SA residues, plays an essential role as a cellular uptake promoter. This hypothesis was further verified by a competitive inhibition assay in which free PBA and free SA were able to reduce the internalization of PLT/DOX NPs but had no influence on LT/DOX NPs (Figure 2A,B and Figure S9, Supporting Information). Though PBA has some advantages, this molecule remains unsatisfactory because of its distinct off‐target effects owing to the distribution of SA residues in normal tissues, especially in the liver.^37^ Figure 2C and Figure S12 (Supporting Information) show that Fru could shield the adjacent hydroxyl group of PBA to significantly reduce the cellular uptake efficiency of PLT NPs at pH 7.4, while PBA regained its targeting ability at pH 6.5 because of the cleavage of Fru. Moreover, the binding of PBA and SA was not affected by the pH, which indicated that PBA could target SA residues in the acidic tumor microenvironments (Figure 2C). As shown in Figure S12 (Supporting Information), the cellular uptake profile of NPs was time‐dependent and the “Fru‐blocking” effect worked as early as 0.5 h.

### 2.5 Endosomal/Lysosomal Process

The endocytic pathway is the major uptake mechanism of tumor cells. After the NPs are internalized, endosomal/lysosomal entrapment becomes the main barrier to the nuclear transportation of DOX. To track the intracellular behavior of the NPs, B16F10 and HepG2 cells were observed by confocal laser scanning microscopy (CLSM) after incubation with PLT/DOX NPs for 2 h and simultaneous staining of the lysosomes with LysoTracker Red. PLT/DOX NPs and lysosomes exhibited a high degree of overlap in B16F10 and HepG2 cells (visible as yellow fluorescence, Figure 2D and Figure S15, Supporting Information), but there was also a strong signal in the cell nuclei. The enhanced endonuclear signal was a result of the acidic pH and high esterase activity in lysosomes, which expedited the dissociation of the ester bond between LMWH and TOS and promoted the release of DOX into the cytoplasm.^44^

### 2.6 In Vitro Inhibitory Effect on Cell Migration and Invasion

**Figure 3:**
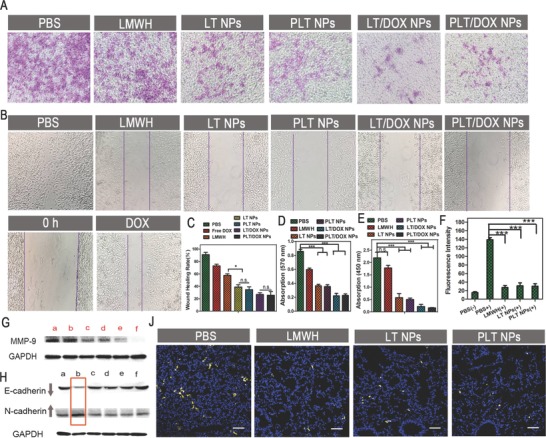
Inhibitory effect of NPs on B16F10 cell migration and invasion in vitro and anti‐implantation effect in vivo. A) Images and D) quantitative analysis of invaded B16F10 cells after separate incubation with PBS, LMWH, LT NPs, PLT NPs, LT/DOX NPs, and PLT/DOX NPs for 48 h. The invaded cells were stained with crystal violet (means ± SD, *n* = 3). *** indicates *p* < 0.001. B) Images and C) healing rate from the wound healing assay after separate incubation with free DOX, LMWH, LT NPs, PLT NPs, LT/DOX NPs, and PLT/DOX NPs for 24 h (means ± SD, *n* = 3). * indicates *p* < 0.05. E) Detection of MMP‐9 in B16F10 cell culture medium by ELISA after separate incubation with PBS, LMWH, LT NPs, PLT NPs, LT/DOX NPs, and PLT/DOX NPs for 30 h (means ± SD, *n* = 3). *** indicates *p* < 0.001. F) The fluorescence intensity of platelets adhering to B16F10 cells in vitro. + indicates coincubation with calcein‐AM labeled platelets, – indicates no coincubation with calcein‐AM labeled platelets (means ± SD, *n* = 3). *** indicates *p* < 0.001. G) Expression of MMP‐9 in B16F10 cells tested by Western blot after separate incubation with (a) PBS, (b) LMWH, (c) LT NPs, (d) PLT NPs, (e) LT/DOX NPs, and (f) PLT/DOX NPs for 30 h. H) Detection of E‐cadherin and N‐cadherin in B16F10 cell culture medium by Western blot after separate incubation with (a) PBS−, (b) PBS+, (c) LMWH+, (d) LT NPs+, (e) PLT NPs+, and (f) PLT/DOX NPs+ for 30 h, + means coincubation with platelets after administration. J) CLSM images of the frozen sections of lungs. The implanted B16F10 tumor cells were identified by CFSE staining (yellow). Cell nuclei were stained with DAPI (blue). The scale bar indicates 200 µm.

Tumor cell migration and invasion are directly associated with metastasis.^45^ B16F10 cells, which exhibit high motility and possess strong pulmonary metastatic tendency, were used to investigate the antimetastatic effect of both blank and DOX‐loaded NPs. For the wound‐healing assay, **Figure** **3**B shows that scratch gaps in PBS groups almost disappeared after 24 h, with a healing rate of ≈90 ± 3.6%. However, the gap‐healing rate decreased to ≈35 ± 3.5 and 31 ± 4.5% after treatment with blank LT NPs and PLT NPs, respectively (Figure 3C). Some studies have reported that LMWH and TOS could reduce the motility of several invasive tumor cells.^25^, ^26^, ^27^, ^28^ The results of the wound‐healing assay indicated that even blank NPs were able to inhibit the motility of B16F10 cells, and both LMWH and TOS retained the ability to inhibit migration after conjugation. Notably, LT/DOX NPs and PLT/DOX NPs exhibited the strongest inhibitory effects on cell motility owing to the loaded DOX (LT/DOX NP groups, 27 ± 2.6%; PLT/DOX NP groups, 25 ± 3.4%). This result indicated that the DOX‐loaded NPs exhibited a greater inhibition of the motility of B16F10 cells than did the blank NPs.

In addition, the invasion inhibitory effect of NPs was assessed by cell invasion assays. Compared with the PBS groups, the number of B16F10 cells that penetrated the Matrigel‐coated membrane was ≈69.7 ± 3.7% in the LMWH‐treated groups. The slight inhibition of invasion might be a result of the cell motility inhibition capacity of LMWH. Interestingly, the blank LT NP‐treated and PLT NP‐treated groups exhibited strong inhibition of invasion, reducing the invasion rate to 42.4 ± 2.6% and 41.3 ± 3.3%, respectively (Figure 3A,D), which indicated that TOS plays an essential role as a powerful inhibitor of cell invasion. In the invasion assays, the tumor cells secreted enzymes such as matrix metalloproteinases, which could degrade the Matrigel and facilitate invasion across the membrane. Some studies have reported that TOS might inhibit the production of matrix metalloproteinases such as MMP‐9, which is directly associated with tumor metastasis.^26^, ^27^, ^28^ To investigate the invasion inhibitory mechanism of TOS, we conducted ELISA to detect the MMP‐9 in culture media and intracellular MMP‐9 produced by B16F10 cells after treatment with PBS, LMWH, LT NPs, PLT NPs, LT/DOX NPs, or PLT/DOX NPs. Figure 3E shows that the concentrations of MMP‐9 in the culture media were reduced in the groups treated with blank NPs and DOX‐loaded NPs (*P* < 0.001), which indicated that the TOS present in NPs could inhibit the production of MMP‐9 in B16F10 cells. The expression of MMP‐9 in cells was detected by Western blot assay. The results also confirmed the MMP‐9 inhibition efficacy of NPs (Figure 3G). However, LMWH did not significantly inhibit the expression of MMP‐9. In conclusion, both LT NPs and PLT NPs exhibited efficient inhibition of the migration and invasion of B16F10 cells. This inhibitory effect allowed these NPs to act synergistically with DOX to treat invasive cancers, as DOX‐loaded NPs further reduced the invasion rates to less than 25%.

### 2.7 Antimetastatic Effect of NPs Exerted by Inhibiting the Adhesion of Platelets to Tumor Cells

Human blood contains ≈400 billion L^−1^ circulating platelets, which are replaced every week. The abundant platelets significantly assist tumor metastasis.^12^, ^13^, ^14^, ^15^, ^16^ To explore the adhesion between tumor cells and platelets in vitro, platelets were extracted from the blood of male C57BL/6 mice, labeled with calcein AM, and coincubated for 30 min with B16F10 cells treated with PBS, LMWH, LT NPs, or PLT NPs. All animal procedures were performed in accordance with the Guidelines for Care and Use of Laboratory Animals of Sichuan University and approved by the Animal Ethics Committee of Sichuan University. Figure 3F indicates that LMWH markedly reduced the number of adhesive platelets on tumor cells compared with that in the PBS group, as did the blank LT NPs and PLT NPs (*p* < 0.001). Free LMWH exhibited a slightly stronger inhibitory effect on tumor cell–platelet interactions than blank NPs, and this phenomenon might be due to the flexible linear structure of LMWH. Furthermore, the implantation of B16F10 cells in lung tissue in vivo was assessed by an implantation model, and images of the frozen lung sections are shown in Figure 3J. The injection of blank NPs in advance prevented adhesion between platelets and tumor cells, thereby exposing tumor cells to flow shear stress and attacks by immune cells and inhibited the epithelial‐mesenchymal‐like transition (EMT) of tumor cells.^12^ As shown in Figure S17 (Supporting Information), B16F10 cells coincubated with platelets showed obvious morphological changes of EMT. They changed from polygon to spindle, which was beneficial for tumor cells to cross the blood vessels and implant in the distant tissues. In contrast, there is no obvious change in the morphology of LMWH, LT NPs, PLT NPs, and PLT/DOX NPs treated cells. Moreover, two pivotal markers involved in EMT were detected by Western blot. The expression of E‐cadherin, which was related to calcium‐dependent cell–cell adhesion,^46^, ^47^ was inhibited and the expression of N‐cadherin, which was related to mesenchymal cell–cell adhesion,^48^ was increased after coincubated with platelets (Figure 3H). The changes of these two markers contributed to the adhesion between tumor cells and vascular endothelial cells, thereby promoted the cross‐vessel movement of tumor cells. However, the expression of E‐cadherin and N‐cadherin did not change significantly compared with the control group, which indicated that LMWH and NPs containing LMWH segments could inhibit the EMT of B16F10 cells through the inhibition of the adhesion between tumor cells and platelets and the hindrance of the extravasation of tumor cells. Based on the above, the implantation of tumor cells in lung tissue was hindered after treatment with NPs (Figure 3J and Figure S16, Supporting Information). In addition, LT NPs and PLT NPs showed similar inhibition abilities, indicating that the modification of adding PBA to LT NPs did not influence the function of LMWH.

### 2.8 Tumor Targeting by PLT‐Fru NPs in Tumor‐Bearing Mice and Metastasis Targeting by PLT NPs in a Metastatic Mouse Model

**Figure 4:**
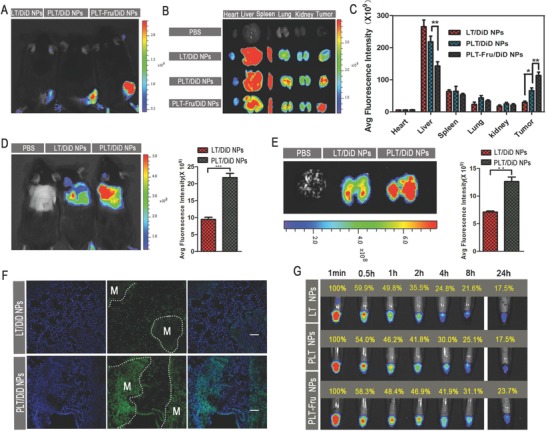
Biodistribution and metastasis targeting capacity of NPs in vivo. A) In vivo images of B16F10 tumor‐bearing mice at 4 h and B) ex vivo images of tumors and organs of tumor‐bearing mice at 24 h after the systemic administration of DiD‐loaded NPs. C) Semiquantitative mean fluorescence intensity results showing the tumor and organ distribution of DiD‐loaded NPs in B16F10 tumor‐bearing mice 24 h after systemic administration (mean ± SD, *n* = 3). * indicates *p* < 0.05, and ** indicates *p* < 0.01. D) In vivo image and average fluorescence intensity semiquantitative results of B16F10 metastasis model mice at 6 h after systemic administration of DiD‐loaded NPs in vivo. *** indicates *p* < 0.001. E) Ex vivo image and average fluorescence intensity semiquantitative results of lungs from B16F10 metastasis mouse model at 6 h after systemic administration of DiD‐loaded NPs. ** indicates *p* < 0.01. F) Confocal images of lung sections from B16F10 metastasis model mice at 6 h after systemic administration of DiD‐loaded NPs (green). Cell nuclei were stained with DAPI (blue). M indicates the metastases in lung. The scale bar represents 200 µm. G) The circulation profile of LT NPs, PLT NPs, and PLT‐Fru NPs in the blood. Cy7‐labeled NPs were used for visualization. Blood was drawn from C57BL/6 mice after tail vein injection with different formulations at each time point and imaged under a fluorescence imaging system.

To investigate the in vivo tumor‐targeting ability of PLT‐Fru NPs, we established a tumor‐bearing mouse model with B16F10 cells inoculated subcutaneously into the backs of C57BL/6 mice. LT NPs, PLT NPs, and PLT‐Fru NPs labeled with the fluorescent probe DiD were injected into different groups of mice through the tail vein. The time‐dependent biodistribution of DiD‐loaded LT NPs, PLT NPs, and PLT‐Fru NPs was measured by an IVIS instrument. Among all the time points, the fluorescence signal peaked at 4 h, indicating rapid accumulation at the tumor site (**Figure** **4**A and Figure S18, Supporting Information). The fluorescence intensity of the PLT NP group in tumor was higher than that of the LT NP group, probably owing to the binding of PBA with SA residues that were overexpressed on the tumor cell membranes. Nevertheless, SA residues were also distributed in normal tissues and caused off‐target effects (Figure 4B,C). Accordingly, we tried to combine PBA with Fru to construct a pH‐sensitive PBA‐Fru structure. PBA‐Fru remained stable in the physiological microenvironment of normal tissue (pH ≈7.4, Figure 1H). In contrast, Fru was easily detached in the acidic microenvironment of tumor tissue (pH ≈6.5), and the affinity of PBA to SA residues was recovered. The fluorescence signals suggested greater accumulation of PLT‐Fru NPs in tumor tissue at all time‐points (2.3‐fold at 4 h) than that of PLT NPs (Figures S18 and S19, Supporting Information). Then, the mice were sacrificed, and ex vivo fluorescence images of the excised organs were obtained. The fluorescence intensity of the PLT‐Fru NP group decreased to ≈66% of that of the PLT group in the liver but was 1.7 times as high as that of the PLT NP group in tumor tissue (Figure 4B,C). The results indicated that the “Fru‐blocking” strategy to reduce the off‐target effect was effective.

The metastasis‐targeting ability of PLT NPs was investigated in the B16F10 metastatic mouse model. IVIS images were taken 6 h after systemic administration of DiD‐loaded NPs. The fluorescence intensity in the lung metastases of the PLT NP group was 1.8 times as strong as that in the LT NP groups (Figure 4D,E). This result was confirmed in the frozen lung tissue sections (Figure 4F and Figure S21, Supporting Information), indicating more efficient metastasis‐targeting ability of PLT NPs.

### 2.9 Circulation Profile of Cy7‐Labeled NPs and Pharmacokinetics Assay

The circulation profile of the NPs was investigated using the Cy7 probe. Interestingly, the results showed that the fluorescence signal of PLT‐Fru/Cy7 NPs was significantly stronger than that of LT/Cy7 NPs and PLT/Cy7 NPs from 4 to 24 h. The fluorescence signal of PLT‐Fru/Cy7 NPs in the blood remained relatively high (23.7%) at 24 h and was 35.4% higher than that of LT/Cy7 NPs and PLT/Cy7 NPs (both 17.5% at 24 h) (Figure 4G). This difference might exist because of the decreased binding between PBA and SA residues in normal tissue achieved by the “Fru‐blocking” strategy. Meanwhile, according to the NP characterization, the negative potential of LT NPs was much lower than that of PLT NPs. This difference in negative potential might explain the lower fluorescence signal of LT/Cy7 NPs than that of PLT/Cy7 NPs within 8 h. The results suggested that the “Fru‐blocking” strategy could prolong the circulation lifetime of the NPs in blood. In addition, the drug concentration–time curve and pharmacokinetic parameters indicated that the area under the curve AUC(0–∞) of DOX in PLT‐Fru/DOX NPs (5.612 mg L^−1^ h) was higher than that in PLT/DOX NPs (3.917 mg L^−1^ h), and the *t*_1/2β_ of DOX was prolonged by ≈54% by PLT‐Fru NPs compared with that observed with PLT NPs (Table S3, Supporting Information).

### 2.10 Antitumor Treatment In Vivo

**Figure 5:**
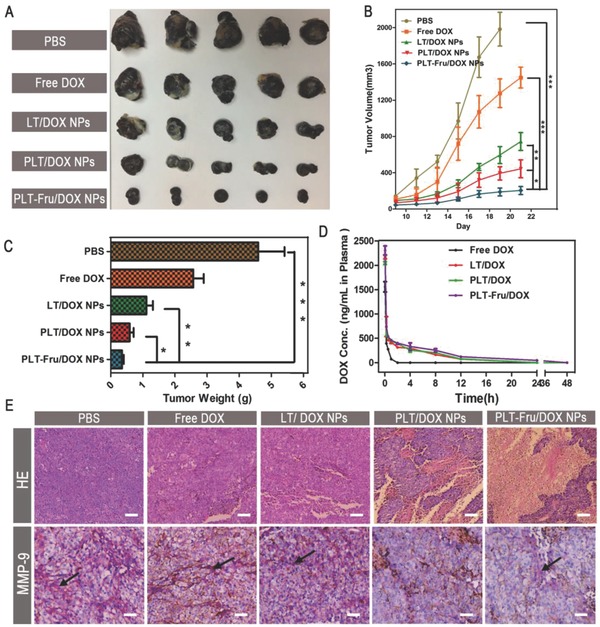
In vivo B16F10 solid tumor treatment. A) Images of B16F10 tumors harvested from C57BL/6 mice administered PBS, free DOX, LT/DOX NPs, PLT/DOX NPs, or PLT‐Fru/DOX NPs (DOX 3 mg kg^−1^) by intravenous injection. B) Tumor volume changes in B16F10 tumor‐bearing C57BL/6 mice during the respective therapies (means ± SD, *n* = 5). * indicates *p* < 0.05,** indicates *p* < 0.01, *** indicates *p* < 0.001. C) Weights of the harvested B16F10 tumors (means ± SD, *n* = 5). * indicates *p* < 0.05, ** indicates *p* < 0.01, *** indicates *p* < 0.001. D) DOX plasma concentration–time profiles in C57BL/6 mice treated with different DOX formulations (at a dose of 3 mg kg^−1^ DOX; *n* = 3). E) Histological analysis of hematoxylin and eosin (H&E) assays and immunohistochemical analysis of MMP‐9 staining for B16F10 tumors. The scale bar indicates 200 µm. The secreted MMP‐9 was stained violet and indicated by black arrows, and the scale bar represents 100 µm.

To investigate the in vivo antitumor efficacy of PLT‐Fru/DOX NPs, B16F10 cells were inoculated subcutaneously into the backs of C57BL/6 mice to establish a tumor‐bearing mouse model. The mice were sacrificed on the 21st d. Then, the tumors were carefully excised from the mice and weighed. As shown in **Figure** **5**A,B, the mice treated with PBS and free DOX exhibited rapid tumor growth. In addition, owing to the effectiveness of PBA in improving tumor targeting and accumulation, the average tumor weight of the LT/DOX NP group was 1.9 times higher than that of the PLT/DOX NP group after treatment. Compared with the other groups, the PLT‐Fru/DOX NP group exhibited maximal antitumor efficiency; the average tumor weight was 1.7 times higher than that of the PLT‐Fru/DOX NP group (Figure 5C). The results suggested that the “Fru‐blocking” strategy successfully retained the pH‐activated affinity of PLT/DOX NPs to SA residues in the acidic tumor microenvironment.

### 2.11 Histological and Immunohistochemical Analysis of Tumors

Tumor tissues were sliced and stained with hematoxylin and eosin (H&E) for histological analysis. While tumor tissues treated with free DOX or LT/DOX NPs retained their complete microstructures, extensive tissue necrosis was observed in the PLT/DOX NP and PLT‐Fru/DOX NP groups. The PLT‐Fru/DOX NP group exhibited a larger area of necrosis than did the PLT/DOX NP group (Figure 5E), indicating a more efficient antitumor effect of the former than that of the latter. In addition, immunohistochemical analysis indicated that the administration of NPs could reduce the secretion of MMP‐9 in the melanoma microenvironment, and due to the improved tumor accumulation of PBA‐modified NPs, the PLT/DOX NP, and PLT‐Fru/DOX NP groups had less MMP‐9 at the tumor site (Figure 5E). By inhibiting the expression of MMP‐9, the self‐delivering micellar NPs may decrease the number of CTCs that penetrate blood vessels from the solid tumor and thereby restrain tumor metastasis. Furthermore, the body weights of the mice were measured every 2 d during the therapy process. The body weights of all the groups remained stable during treatment. Thus, we suggest that no obvious systemic toxicity was observed at the dose used (Figure S22, Supporting Information).

### 2.12 Preliminary Safety Evaluation

The mice organs were sliced and stained with H&E using the same method as described above. There was no morphological difference between the experimental groups and the control group after treatment except for the free DOX‐treated group, which exhibited distinct cardiotoxicity (Figure S23, Supporting Information). Hematological analysis was conducted to evaluate the toxicities of different preparations. As shown in Figure S24 (Supporting Information), significant downward trends of WBC (white blood cell, dropped by nearly 52%) and RBC (red blood cell, dropped by nearly 20%) counts were observed in the free DOX‐treated group compared with the PBS group. This result suggested that DOX might have side effects on the immune system. However, mice treated with PLT/DOX NPs did not exhibit a significant reduction of the three types of blood cells, which indicated that the nanosystem could reduce the toxicity of DOX. LMWH and blank NPs had no clear effect on the number of blood cells at this dosage. A biochemical analysis of the serum was also conducted to evaluate organ toxicity. Free DOX significantly increased the levels of ALT (alanine aminotransferase), AST (aspartate trans‐aminase), and CK (creatine kinase), which indicated that free DOX could cause liver injury and heart injury. PLT/DOX NPs could reduce the occurrence of these organ injuries. LMWH, blank, and DOX‐loaded NPs exhibited no distinct kidney injury at this dosage. Moreover, we also focused on the preliminary safety of the “Fru‐blocking” strategy. As shown in Figure S25 (Supporting Information), the PLT‐Fru/DOX NPs did not cause severe toxicity. The preliminary safety evaluation demonstrated that LT NPs and PLT NPs were biocompatible and had clinical transformation potential.

### 2.13 Antimetastatic Treatment In Vivo

**Figure 6:**
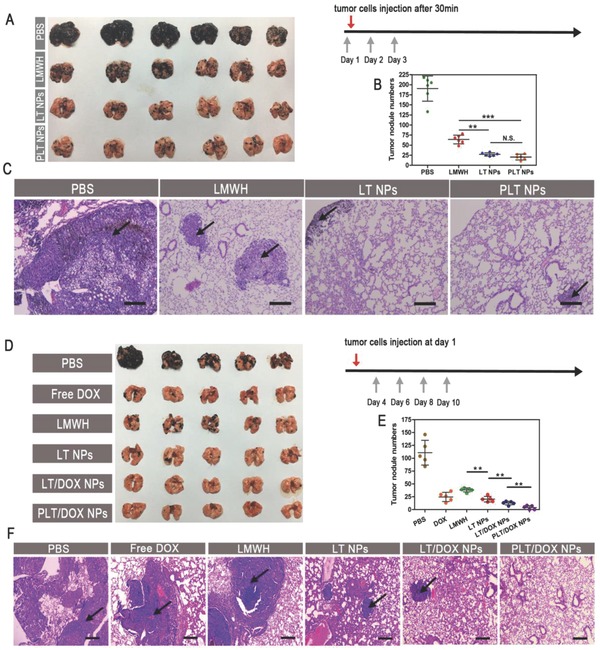
In vivo antimetastasis treatment. A) Images of lungs, B) the number of B16F10 metastases on the lung surface, and C) histological analysis of H&E assays of lungs from B16F10 metastasis mouse model after pretreatment with PBS, free LMWH, LT NPs, or PLT NPs by intravenous injection (at doses of 60 mg kg^−1^) (means ± SD, *n* = 6). ** indicates *p* < 0.01 and *** indicates *p* < 0.001. The scale bar indicates 100 µm. D) Images of lungs, E) the number of B16F10 metastases on the lung surface, and F) histological analysis of H&E assays for lungs from B16F10 metastasis mouse model after treatment with PBS, free DOX, free LMWH, LT NPs, LT/DOX NPs, or PLT/DOX NPs by intravenous injection (at equivalent dose of 2.5 mg kg^−1^ DOX) (means ± SD, *n* = 5). ** indicates *p* < 0.01. The scale bar represents 100 µm.

Three tumor metastasis mouse models were established to investigate the metastasis inhibition ability of blank NPs using two tumor cell lines. The treatment started 30 min before the injection of B16F10, CT26, or 4T1 cells to inhibit the adhesion of platelets in advance. The lungs were harvested and photographed at day 20, and the nodules on the surfaces were counted. Obviously, the B16F10 cells easily metastasized to the lungs and occupied a majority of the area of the lung tissue in the PBS group (**Figure** **6**C). Due to the inhibition of platelet–tumor cell adhesion by LMWH and the toxicity of TOS on tumor cells,^18^, ^19^, ^20^ fewer metastatic nodules were observed in the blank LT NP‐treated group than in the LMWH‐treated group (Figure 6A,B and Figures S26 and S27, Supporting Information), which confirmed the synergistic antimetastatic effect of LMWH and TOS. In addition, prolonged circulation of NPs might prolong the adhesion inhibition of LMWH. Thus, the blank NPs exhibited a better antimetastatic effect than free LMWH.

Another dosage regimen was tested to investigate the antimetastatic effect of DOX‐loaded NPs. The treatment started at day 4 after the injection of B16F10 cells. The LMWH and LT NPs also exhibited high antimetastatic effects in this model. Though the free DOX group showed a similar number of nodules to the LT NP group (Figure 6D,E), the mean sizes of the nodules were significantly larger in the free DOX group than in the LT NP group (Figure 6F). The DOX‐loaded groups showed very strong antimetastatic effects because not only was platelet adhesion inhibited (by LMWH) but also the colonizing cells were directly killed (by DOX). Additionally, PBA, with high affinity for SA residues, on NP surfaces increased the targeting distribution of the NPs in metastatic nodules, and the metastatic nodules in the PLT/DOX NP group were almost invisible. The self‐delivering micellar NPs, with strong metastatic nodule‐targeting ability and low toxicity, loaded with the chemotherapeutic drug DOX showed extraordinary antimetastatic effects in a metastatic mouse model of melanoma. Importantly, the higher the metastatic ability of tumor cells is, the more SA residues the cells express.^35^ Furthermore, inhibiting the expression of SA in tumor cells could even reduce lung metastasis.^49^ Based on the above, these PBA‐modified, multiphase, antimetastatic, self‐delivering, micellar NPs were particularly suitable for treating highly invasive cancers.

## 3 Conclusion

In summary, we established a tumor‐microenvironment‐activated, PBA‐modified, self‐delivering, micellar NP that acted as both antimetastatic agent and nanocarrier to treat both solid tumors and metastases. The constituent materials of PLT/DOX NPs included the antimetastatic agents LMWH, TOS, and the chemotherapeutic drug DOX. PLT NPs could inhibit the adhesion between tumor cells and platelets and the production of MMP‐9 to inhibit different phases of the metastasis cascade. The in vivo results showed that not only the DOX‐loaded NPs but also the blank NPs could significantly reduce the number of metastatic nodules without systemic toxicity. Based on the excellent antimetastatic capacity of blank NPs, the application of blank NPs with low toxicity after diagnosis could be effective as a preoperative and postoperative adjuvant therapy or for the prevention of the metastasis of most metastatic tumors, which warrants further research. In addition, the “Fru‐blocked” PBA could be activated specifically at the tumor site. This simple strategy efficiently reduced the off‐target effect and further enhanced NP accumulation at the tumor site. The substantial in vivo curative effects against solid melanomas and metastasis demonstrated the efficacy of this self‐delivering micellar NP. The simple targeting strategy based on the tumor‐microenvironment‐activated PBA is promising for the treatment of various invasive tumors that overexpress SA residues.

## 4 Experimental Section

*Synthesis and Characterization of the LMWH‐TOS Conjugate*: The amphiphilic LMWH‐TOS was synthesized by attaching TOS to LMWH via ester bond formation. First, TOS (175 mg) and the activation agents (N‐(3‐dimethylaminopropyl)‐N′‐ethylcarbodiimide (EDC), 126.5 mg; N‐hydroxysuccinimide (NHS), 76.0 mg; and 4‐diaminomethylpyridine (DMAP), 20.2 mg) were dissolved in N,N‐dimethylformamide (DMF, 10 mL). The mixture was stirred for 3 h under a nitrogen atmosphere at room temperature to activate the carboxylic acid groups in TOS. Meanwhile, LMWH (100 mg) was dissolved in formamide (10 mL) at 50 °C and added slowly into the activated TOS solution. The reaction mixture was stirred for 24 h under a nitrogen atmosphere at room temperature and then dialyzed against deionized water for 72 h and lyophilized. The successful synthesis of the LMWH‐TOS conjugate was confirmed by ^1^H‐NMR spectroscopy.

*Synthesis and Characterization of the PBA‐LMWH‐TOS Conjugate*: PBA‐LMWH was synthesized via amide bond formation. Briefly, LMWH (250 mg) was dissolved in PBS (25 mL) containing the activation agents (EDC, 790.0 mg; NHS, 474.7 mg). The pH of the mixture was adjusted to 6.0, and the mixture was stirred for 0.5 h at room temperature to activate the carboxylic acid groups in LMWH. Then, PBA (15.0 mg), fully dissolved in PBS (2 mL), was added slowly, and the pH of the mixture was adjusted to 8.0. The reaction mixture was stirred for 12 h under a nitrogen atmosphere at room temperature and was then dialyzed against deionized water for 48 h. After lyophilization, the resulting white powder, the PBA‐LMWH conjugate, was stored at 2–8 °C for further experiments. The amphiphilic PBA‐LMWH‐TOS was synthesized by attaching TOS to PBA‐LMWH via ester bond formation. First, TOS (175.2 mg) and the activation agents (EDC, 126.5 mg; NHS, 76.0 mg; and DMAP, 20.2 mg) were dissolved in DMF (10 mL). The mixture was stirred for 3 h under a nitrogen atmosphere at room temperature to activate the carboxylic acid groups of TOS. Meanwhile, the PBA‐LMWH conjugate (100 mg) was dissolved in formamide (10 mL) at 50 °C and added slowly into the activated TOS solution. The reaction mixture was stirred for 24 h under a nitrogen atmosphere at room temperature and then dialyzed against deionized water for 72 h and lyophilized. Successful synthesis of all the conjugates was confirmed by ^1^H‐NMR spectroscopy.

*Quantitative Measurement of PBA and LMWH in the PBA‐LMWH‐TOS Conjugate*: Because PBA contained a fluorophore, a fluorescence standard curve for the quantitative determination of PBA was constructed (*E*_x_ = 302 nm, *E*_m_ = 375 nm). The quantitative standard curve of LMWH was made by using toluidine blue spectrophotometry. In brief, LMWH was dissolved in PBS to prepare LMWH solutions with a series of concentrations (1, 2, 4, 8, 12, and 14 µg mL^−1^). The toluidine blue solution was prepared by dissolving 0.1 g of NaCl and 2.5 mg of toluidine blue in 50 mL of water. Then, 2.5 mL of each of the LMWH solutions were mixed with 2.5 mL of toluidine blue solution individually. After thoroughly vortexing for 0.5 min, 5 mL of *n*‐hexane was added into the former mixture, and the mixture was vortexed for an additional minute. The water layer was isolated by a separatory funnel, and the ultraviolet absorption value was measured at 629 nm.

*Preparation and Characterization of DOX‐Loaded NPs*: DOX was used as a model drug in this investigation. DOX‐loaded NPs were prepared by ultrasonic emulsification. First, DOX hydrochloride (10 mg) was neutralized with triethylamine (7.2 µL) in dichloromethane (5 mL) by stirring overnight in the dark. Then, the reaction mixture was directly mixed with the PBA‐LMWH‐TOS or LMWH‐TOS conjugate (100 mg) and stirred at 37 °C for 3 h. Then, the mixture was added to deionized water (50 mL) under ultrasonication (100 W, 5 s/5 s, 7 min). The resulting emulsion was vaporized by rotary evaporation at 37 °C to remove the dichloromethane. Subsequently, the red solution was dialyzed against deionized water for 3 h and lyophilized after filtration through a 0.22 µm filter. The zeta potentials and mean sizes of the NPs in deionized water were measured by a Malvern Zetasizer Nano ZS90 (Malvern Instruments Ltd., UK). In addition, the morphologies of the NPs were observed under a transmission electron microscope (Hitachi H‐600, Japan).^38^ To measure the drug‐loading capacities of the NPs, the micelle dispersion was disrupted by DMSO (100 volumes), and the drug content was analyzed by a Varioskan Flash multimode reader (Thermo, USA) at *E*_x_ = 488 nm and *E*_m_ = 555 nm. Drug‐loading capacity (%) = (weight of DOX in NPs)/(weight of DOX‐loaded NPs) × 100.

## Conflict of Interest

The authors declare no conflict of interest.

## Supporting information

Supplementary
