# Supplementary material for: Enhanced Melanoma‐Targeted Therapy by “Fru‐Blocked” Phenyboronic Acid‐Modified Multiphase Antimetastatic Micellar Nanoparticles
Source: Adv Sci (Weinh). 2018 Jul 13;5(11):1800229. doi: 10.1002/advs.201800229 (PMC6247072; doi:10.1002/advs.201800229)
Supplement: Supplementary file 1 — Supplementary [file ADVS-5-1800229-s001.pdf]

Copyright WILEY-VCH Verlag GmbH & Co. KGaA, 69469 Weinheim, Germany, 2018.

Supporting Information

for *Adv. Sci.,* DOI: 10.1002/advs.201800229

Enhanced Melanoma-Targeted Therapy by "Fru-Blocked" Phenyboronic Acid-Modified Multiphase Antimetastatic Micellar Nanoparticles

*Yang Long, Zhengze Lu, Ling Mei, Man Li, Kebai Ren, Xuhui Wang, Jiajing Tang, Zhirong Zhang, and Qin He**

Supporting Information

Enhanced Melanoma-targeted Therapy by “Fru-blocked” Phenyboronic Acid-modified Multiphase Anti-metastatic Micellar Nanoparticles

*Yang Long, Zhengze Lu, Ling Mei, Man Li, Kebai Ren, Xuhui Wang, Jiajing Tang, Zhirong Zhang and Qin He**

Key Laboratory of Drug Targeting and Drug Delivery Systems, West China School of Pharmacy, Sichuan University, No. 17, Block 3, Southern Renmin Road, Chengdu 610041, China.

*Corresponding author: Tel/Fax: +86-28-85502532; E-mail: qinhe@scu.edu.cn

Experimental Section

1. Materials. Doxorubicin hydrochloride (DOX · HCl) (> 98%), D-α-tocopheryl succinate (TOS) (> 95%) and low molecular weight heparin (LMWH, MW 3800~5000, Mw/Mn (PDI) = 1.34) was purchased from Melonepharma (Dalian, China). D-fructose was purchased from TCI (Shanghai, China). 3-aminophenylboronic acid (PBA) (> 98%) was purchased from Sigma-Aldrich. N-Hydroxy-succinimide (NHS) (> 98%) and 1-[3-(dimethylamino) propyl]-3-ethylcarbodiimide hydrochloride (EDC) (> 99%) were obtained from J&K Scientific (Beijing, China). 4-(Dimethylamino)pyridine (DMAP) (> 99%) was obtained from Sigma-Aldrich. Matrigel was bought from BD Biosciences (San Jose, CA). 3-(4,5-dimethyl-2-tiazolyl)-2,5-diphenyl-2H-tetrazolium bromide (MTT), 1,1’-dioctadecyl-3,3,3’,3’-tetramethylindodicarbocyanine (DiD), 4,6-diamidino-2-phenylindole (DAPI), LysoTracker Red (DND-99) and calcein-AM were purchased from Beyotime Biotechnology (Shanghai, China). Cy7-NH_2 was purchased from Lumiprobe (USA). carboxyfluorescein succinimidyl amino ester (CFSE) was purchased from Dojindo (Kumamoto,

Japan). Plastic cell culture dishes and plates were purchased from Wuxi NEST Biotechnology Co. (Wuxi, China). Mouse matrix metalloproteinase-9 (MMP-9) elisa kit was purchased from Wuhan ColorfulGene Biological Technology Co., LTD (Wuhan, China). Anti-MMP-9 antibody was purchased from R&D systems (Minnesota, America). Other reagents and chemicals were analytical level. Both anti-E-cadherin and anti-N-cadherin were purchased from Zen Bioscience (Chengdu, China).

2. Cell Lines and Animals. Mouse melanoma cells (B16F10), human liver hepatocellular carcinoma cells (HepG2), mouse breast cancer cells (4T1), mouse colorectal cancer cells (CT26) and the kidney cells of the African Green Monkey (COS-7) were purchased from the Shanghai Institutes for Biological Sciences, CAS (SIBS, Shanghai, China). All cells mentioned above were cultured in Dulbecco's modified Eagle's medium (DMEM, GIBCO) supplemented with 10% FBS (HyClone), 100 μg/mL streptomycin and 100 U/mL penicillin at 37 °C in a humidified atmosphere of 5% CO_2 in air. C57BL/6 male mice and Balb/c male mice (about 5-week-old, 18-22 g, SPF) were purchased from Dashuo Experimental Animal Company (Chengdu, China). **All animal experiments were performed according to the rules of Experimental Animals Administrative Committee of Sichuan University.**

3. Serum Stability Assay A serum stability assay was performed to evaluate the stability of different NPs in serum. Samples were mixed with FBS (formulation: FBS (v/v) = 9:1 and 1:1) respectively (final material concentration, 1 mg/mL). The mixture was added to a 96-well plate and incubated in a shaking bed at 37 °C with gently oscillation for 24 h. The absorbance of the mixture was measured at 750 nm using a Varioskan Flash Multimode Reader (Thermo, USA) at different time points (0, 1, 2, 4, 8, 12, 24 h).

4. *In Vitro* Hemolysis Assay. To investigate the interactions of different NPs with blood erythrocytes, we performed hemolysis assays as described previously with modifications.[1] Different concentrations of PLT NPs were incubated with 2% RBC suspensions from C57BL/6

mice for predetermined periods (1, 2, 4, and 8 h) at 75 rpm and 37 °C. Then, each sample was centrifuged at 1500 rpm for 5 min at each time point. The absorbance of the supernatant was measured at 540 nm using a Varioskan Flash multimode reader (Thermo, USA). PBS and the RBC suspension treated with 0.2% Triton X-100 were used as negative and positive control groups, respectively. The hemolysis percentage was calculated according to the following formula:

$$\text{Hemolysis (\%)} = (A_{sample} - A_{PBS} / (A_{Triton\text{-}100} - A_{PBS})) \times 100\%.$$

5. *In Vitro* Drug Release. The *in vitro* DOX release behavior of LT/DOX NPs and PLT/DOX NPs was evaluated. Aliquots (1 mL, at equivalent dose of DOX 100 μg) of NP dispersions were loaded into dialysis bags (7 kDa molecular weight cutoff). Each dialysis bag was immersed in PBS (50 mL, pH 7.4 for LT/DOX NPs and pH 5.0, 6.5, or 7.4 for PLT/DOX NPs, adjusted with phosphoric acid) and incubated in a shaking bed at 75 rpm and 37 °C. Aliquots (200 μL) of the release media were collected at predetermined time points (1, 2, 4, 6, 8, 24 and 48 h) while the same volume of fresh medium was replenished. The amount of released drug was determined using a Varioskan Flash Multimode Reader (Thermo, USA) at Ex=488 nm and Em=555 nm

6. pH-sensitive Affinity Assay and Combination Stability Assay of PBA-Fru. A series concentrations of D-fructose (Fru) solution, sialic acid (SA) solution and 3-aminophenylboronic acid (PBA) solution were prepared using pH 6.5 and pH 7.4 phosphate buffer solution (PBS) respectively (Fru: 5, 10, 20, 40, 80, 160, 320 mM, SA: 0.45, 0.9, 1.8, 3.6, 7.2, 14.4, 28.8 mM, PBA: 120 μM). Fru (or SA) solution and PBA solution (V/V = 1:1) of the same pH value was mixed and incubated at 37 °C and 75 rpm for 1 h, and the fluorescence intensity was measured by a Varioskan Flash Multimode Reader (Thermo, USA; Ex = 302 nm and Em = 375 nm). The combination stability assay of PBA-Fru (Fru: 20 mM, PBA: 60 μM) was carried out using the same method. The mixture was added in a 96-well plate (200 μL per well) and incubated at 37 °C and 75 rpm. The fluorescence intensity was measured at each time point (20, 40 min, 2, 4, 7, 12, 24, 36, 72 h).

7. *In Vitro* Cytotoxicity Analysis. The cytotoxicities of different formulations were evaluated in B16F10 cells by the MTT assay. Briefly, B16F10 cells were seeded in 96-well plates (3×10^3 cells/well) and incubated for 12 h at 37 °C and in a 5% CO_2 atmosphere. The culture medium was removed, and then, free DOX, LT NPs, PLT NPs, LT/DOX NPs or PLT/DOX NPs were added at a series of concentrations and incubated with the cells for an additional 48 h. Then, 20 μL of MTT solution (5 mg/mL in PBS) was added to each well, and the plates were incubated for 4 h at 37 °C and in a 5% CO_2 atmosphere. Finally, the culture medium was removed, and the residue was dissolved in 150 μL of DMSO per well. The absorbance of each group was measured by a Varioskan Flash multimode reader (Thermo, USA) at 570 nm. The cell viability (%) was calculated by the following formula: Cell viability (%) = $A_{test} - A_{DMSO} / A_{control} - A_{DMSO} \times 100$.

8. Cellular Uptake and Intracellular Delivery in B16F10 Cells. To investigate the cellular internalization of different micelles, uptake assays using SA-overexpressing B16F10 cells and HepG2 cells and SA-underexpressing COS-7 cells as the recipient cells were conducted. We conducted the cell uptake assays in pH 7.4 and pH 6.5 serum-free media. Briefly, B16F10 cells were seeded in 6-well plates (3×10^5 per well) and grown overnight before incubation with DiD-loaded NPs. After incubation with LT/DiD NPs, PLT/DiD NPs, PLT-Fru/DiD NPs or PLT-SA/DiD NPs in pH 6.5 or pH 7.4 medium for 2 h, the cells were harvested and washed with PBS. After centrifugation (3000 rpm for 3 min), the cells were resuspended in PBS (300 μL), and the mean fluorescence of DiD in individual cells was quantified by a flow cytometer (FL4 675 nm, Beckman Coulter, Inc., USA, Cytomics FC 500).

We also used CLSM to detect the fluorescence signals of different cells treated with different preparations. Three types of cells were cultured on coverslips in 6-well plates (2×10^5 per well) as described above. To verify the interaction between PBA and SA residues, free SA (500 μM) was pre-incubated with micelle suspensions for 2 h, and the mixture was added to the cells. Meanwhile, PBA (500 μM) was pre-incubated with cells for 1 h prior to the addition of the DOX-loaded NP suspensions to the cells, respectively. The cells were incubated for 2 h, washed with PBS (pH 7.4)

for three times, and fixed in 4% (m/v) paraformaldehyde solution for 10 min. Then, the cells were dried under an air stream and stained with 0.5% 4′,6-diamidino-2-phenylindole (DAPI) for 4 min. After being washed three times with PBS, the cells were mounted (Vectashield antifade mounting medium H-1000) and observed by CLSM (DOX: E_x = 488 nm, E_m = 555 nm; DAPI: E_x = 340 nm, E_m = 488 nm; Olympus Co., Ltd, FV1000, Japan).

To track intracellular NP delivery, B16F10 cells and HepG2 cells were stained with LysoTracker Red DND-99 (E_x = 577 nm, E_m = 590 nm) 30 min before the end of the cellular uptake assay and then photographed by CLSM using the staining and mounting method described above.

9. Cellular Uptake by HepG2 and COS-7 Cells. To further investigate the cellular internalization of different NPs, cell uptake assays using HepG2 cells overexpressing SA residues and COS-7 cells with low expression of SA residues were carried out. Briefly, the two cell lines were seeded in 6-well plates (3×10^5 per well) individually and grown overnight before being incubated with DOX-loaded NPs. After treatment with PBS, free DOX, LT/DOX NPs or PLT/DOX NPs (at equivalent doses of 10 μg mL^{-1} DOX) for 0.5 h, 1 h and 3 h, the cells were harvested and resuspended with PBS (300 μL), and the mean fluorescence of DOX in individual cells was quantified by a flow cytometer (Beckman Coulter, Inc., USA, Cytomics FC 500).

10. *In Vitro* Inhibitory Effect on Cell Migration and Invasion. The inhibitory effects of LT/DOX NPs and PLT/DOX NPs on the migration and invasion of B16F10 cells were evaluated by wound-healing and Transwell assays. For the wound-healing assay, cells were seeded into 6-well plates and allowed to grow to 90-95% confluency. Scratch wounds were generated with a 200-μL pipette tip, and then, the cells were washed twice with PBS to form cell-free wounds. Then, the cells were incubated with blank medium (as a control), free DOX, LT NPs, PLT NPs, LT/DOX NPs or PLT/DOX NPs at predetermined concentrations. After incubation with different formulations, images were obtained at 0 h and 24 h with an inverted microscope. The wound-healing rate was calculated according to the previously described method. For the cell invasion assay, 1×10^5 cells

were added to the upper chambers of the Transwells (24-well insert; pore size, 8 μm; Corning, USA) coated with Matrigel (BD Biosciences). In this assay, 100 μL of serum-free medium with PBS, LMWH, free DOX, blank LT NPs, PLT NPs (40 μg mL^{-1}), LT/DOX NPs or PLT/DOX NPs (at an equivalent dose of 0.5 μg mL^{-1} DOX) was loaded into the upper chambers, while the lower chambers were filled with 600 μL of culture medium containing 20% FBS as a chemoattractant. After 48 h of incubation, the migrated or invaded cells were fixed in 4% (m/v) paraformaldehyde solution for 10 min and stained with crystal violet. Finally, the bottoms of the chambers were viewed under an inverted microscope in five predetermined fields. Then, the crystal violet was eluted by a 33% acetic acid solution, and the absorbance was measured at 570 nm using a Varioskan Flash multimode reader.

11. *In vitro* MMP-9 Expression Detection. In brief, B16F10 cells were seeded in 6-well plates (1.5 × 10^{5} per well) individually and grown overnight before incubating with NPs. Afterwards cells were treated with PBS, LMWH, LT NPs, PLT NPs, LT/DOX NPs or PLT/DOX NPs (at an equivalent dose of 100 μg mL^{-1} PLT NPs) for 30 h. Then, the culture medium of each group was analyzed using mouse MMP-9 ELISA kit. Moreover, MMP-9 in cells was detected by Western blot assay. Cells in each well were harvested and mixed with 50 μL cell lysate buffer (Beyotime, China). Samples were centrifuged at 4°C and 13,000 rpm for 15 min, and the supernatant was collected as Western blot assay samples. Each sample was then separated by 6% SDS-PAGE, and followed by transferring to polyvinylidene difluoride membranes. Finally, samples were incubated with antibody. HRP-labeled mouse anti-goat secondary antibody was added and measured using a Bio-Rad ChemiDoc MP System.

12. Adherence of Platelets to Tumor Cells *In Vitro* and Implantation in Lung Tissue of B16F10 Cells *In Vivo*. Platelet-rich plasma (PRP) was obtained from male C57BL/6 mice and washed with PBS and then labeled with 5 μM calcein AM for 20 min using a previously reported method.[2] Meanwhile, the B16F10 cells were seeded in 6-well plates and grown to approximately 80% confluency. Then, the medium was changed, and a total of 1 × 10^{6} fluorescein-labeled platelets

were added to each well. After treatment with PBS (as a control), free LMWH, LT NPs or PLT NPs, the cells were washed three times with PBS. Finally, the cells were lysed with DMSO, and the fluorescence signal was detected by a fluorospectro-photometer at E_x = 490 nm and E_m = 515 nm.

To investigate the efficacy of NPs against CTC implantation, C57BL/6 mice were pretreated with PBS, free LMWH, LT NPs or PLT NPs (60 mg kg^{-1}) 30 min in advance. Meanwhile, the B16F10 cells were stained with carboxyfluorescein succinimidyl ester (CFSE) (20 μM, 37 °C, 15 min). Then, 2 × 10^5 CFSE-labeled B16F10 cells were injected into mice via the tail vein. The lungs were harvested 30 min after cell injection and frozen section analysis was performed. Cell nuclei were stained with DAPI. The distribution of tumor cells in the lungs was observed by CLSM (the fluorescence signal of CFSE was detected at E_x = 496 nm and E_m = 516 nm).

13. *In vitro* E-cadherin and N-cadherin Expression Detection. Platelet-rich plasma (PRP) was obtained from male C57BL/6 mice. B16F10 cells were seeded in 6-well plates (1.5 × 10^5 per well) individually and grown overnight before incubating with NPs and platelets. After treated with PBS-, PBS+, LMWH+, LT NPs+, PLT NPs+, PLT/DOX NPs+ (at an equivalent dose of 50 μg mL^{-1} PLT NPs; + means co-incubation with platelets at 30 minutes after administration) for 30 h, protein samples were prepared and analyzed according to the above method of Western blot assay.

14. *In Vivo* Tumor-targeting Assay and Metastasis-targeting assay. The B16F10 solid tumor model was generated by inoculating 1 × 10^6 B16F10 cells on the right sides of the backs of male C57BL/6 mice. *In vivo* imaging and biodistribution experiments were performed on the tumor-bearing C57BL/6 male mice on the 13th day after the injection of B16F10 cells, by which time the tumors had grown to an approximate diameter of 1 cm. The NPs were labeled with DiD via physical entrapment using the method described above for DOX entrapment. Briefly, the LT conjugate or PLT conjugate was added to DiD dissolved in dichloromethane and stirred at 37 °C for 3 h. Then, the mixture was added to five volumes of PBS, sonicated and purified. The tumor-bearing C57BL/6 mice were injected with PBS, LT/DiD NPs, PLT/DiD NPs or PLT-Fru/DiD NPs (NPs : Fru = 1 : 1,

w/w, 20 μg DiD per mouse) through the tail vein. Then, the tumor regions were imaged using an *in vivo* imaging system (IVIS Lumina Series III, PerkinElmer, USA) at predetermined time points (1, 4, 8, and 24 h). The mice were sacrificed at 24 h, and the tumors as well as major organs, including the heart, liver, spleen, lungs, and kidneys, were collected and subjected to *ex vivo* imaging. Then, the distribution of NPs in the tumor site was observed by CLSM of frozen sections.

For the metastasis-targeting assay, a total of 2 × 10^5 B16F10 cells in 100 μL of PBS were injected into C57BL/6 mice via the tail vein to generate a lung metastatic mouse model of melanoma. On the 15th day after the injection of B16F10 cells, the mice were injected with PBS, DiD-loaded LT NPs or PLT NPs (20 μg DiD per mouse) through the tail vein. *In vivo* images of the B16F10 metastasis model mice were taken at 6 h after the systemic administration of DiD-loaded NPs. Then, the mice were sacrificed, the mouse lungs were harvested, and *ex vivo* fluorescence images of the lungs were obtained using the IVIS instrument.

15. Circulation Profile of Cy7-labeled NPs and Pharmacokinetic Assay. To investigate the circulation profiles of the NPs, we labeled the NPs with the fluorescent probe Cy7. In brief, Cy7-NH_2 was connected to LMWH via an amide bond. C57BL/6 mice were injected with LT/Cy7 NPs, PLT/Cy7 NPs or PLT-Fru/Cy7 NPs (equivalent to 30 μg of Cy7 per mouse) through the tail vein. Approximately 60 μL of blood was collected in heparin-treated tubes from the orbital sinus at each time point (1 min, 0.5, 1, 2, 4, 8, 24 h), and 40 μL of blood from each sample was transferred into a plastic PCR tube (Axygen). The fluorescence intensity of each blood sample was observed and determined by an IVIS instrument. The fluorescence intensity in each group was normalized by considering the intensity at 1 min to be 100%.

For the pharmacokinetic assay, male C57BL/6 mice (6 mice/group) were injected with free DOX, LT/DOX NPs, PLT/DOX NPs or PLT-Fru/DOX NPs (at an equivalent dose of 3 mg kg^{-1} DOX) through the tail vein. Three mice were randomly chosen for the first 5 time points, and the other 3 mice were used for the remaining 5 time points. Approximately 100 μL of blood was

collected in 0.5-mL heparin-treated tubes from the orbit sinus at each time point (3, 15, and 30 min and 1, 2, 4, 8, 12, 24, and 48 h). The blood samples were centrifuged at 4 °C and 5,000 rpm for 10 min to obtain 40 μL of supernatant plasma, and four volumes of acetonitrile (160 μL) was added to each sample. The samples were oscillated for 3 min, sonicated for 15 min, and centrifuged at 4 °C and 12,000 rpm for 10 min. The supernatant was collected and filtered through a 0.22-μm filter for LC-MS/MS analysis (Agilent Technologies 6420 triple quadrupole LC/MS). The chromatographic conditions were as follows: chromatographic column, C18 (2.1 × 50 mm, 1.8 μm); mobile phase, acetonitrile:0.1% formic acid in water (V/V = 28:72). The mass spectrometry conditions were as follows: an electrospray ionization source was used as the ion source; multiple reaction monitoring (MRM) with the transitions of m/z 544.2 → m/z 397.2 and electrospray ionization in positive mode were used to quantify DOX. The sample size was 10 μL/sample.

16. *In Vivo* Antitumor Assay. The B16F10 solid tumor model was generated by inoculating 1×10^6 B16F10 cells on the right sides of the backs of C57BL/6 mice. Then, the B16F10 tumor-bearing C57BL/6 male mice were randomly divided into 5 groups (n = 6), and the treatment was started on the 9th day after tumor implantation. The mice were intravenously injected with PBS, free DOX, LT/DOX NPs, PLT/DOX NPs or PLT/DOX NPs pre-incubated with Fru (PLT-Fru/DOX NPs, NPs:Fru = 1:1, w/w) 2 h in advance (at an equivalent dose of 3 mg kg^{-1} DOX, conjugates:DOX = 19:1, w/w). Each formulation was administered every 2 days for a total of 5 times. Tumor growth and body weight were recorded and calculated. The tumor volume was calculated using the formula ($L \times W^2 / 2$), where W was the smaller diameter and L was the larger diameter. On the 21st day, the mice were sacrificed, and the tumors and major organs were collected for *ex vivo* histological analysis. The tumor inhibition ratio (TIR) was calculated using the equation TIR (%) = $(1 - V_t / V_c) \times 100$, where V_c and V_t were the average tumor volumes at day 21 in the control and treatment groups, respectively.

17. Preliminary Safety Evaluation. To evaluate the preliminary *in vivo* toxicities of different preparations, healthy male C57BL/6 mice (approximately 5 weeks old, 18-22 g, specific pathogen

free (SPF)) were treated with PBS, free DOX, LMWH, LT NPs, PLT NPs and PLT/DOX NPs (at an equivalent dose of 3 mg kg^{-1} DOX) individually every 2 days for a total of 5 doses. Then, 24 h after the final administration, whole blood was obtained in $EDTAK_2$ anticoagulative tubes for routine blood examination (Mindray, BC-2800Vet, China), and serum was obtained for the biochemical analysis (TECOM, TC6010L, China).

18. *In Vivo* Efficacy of NPs against Lung Metastasis. Three tumor metastasis mouse models were established to investigate the anti-metastatic ability of blank NPs by injecting three tumor cell lines into mice via the tail vein respectively. Briefly, mice were intravenously injected with PBS, free LMWH, LT NPs or PLT NPs (60 mg kg^{-1}) to inhibit the adhesion of platelets in advance, and then, 2×10^5 B16F10 cells, 2×10^5 CT26 cells or 1×10^5 4T1 cells in 100 μL of PBS were injected into the mice via the tail vein after 30 min. The once-daily administration continued for an additional two days. The mice were sacrificed on the 20th day, and the lungs were collected and imaged. Then, the lung tissues were subjected to H&E staining.

We also tested another dosage regimen to investigate the anti-metastatic effect of DOX-loaded NPs. A total of 2×10^5 B16F10 cells in 100 μL of PBS were injected into the C57BL/6 mice via the tail vein to generate a lung metastatic mouse model of melanoma. On the 4th day, the mice were randomly divided into 6 groups and administered PBS, free DOX, free LMWH, blank LT NPs (60 mg kg^{-1}), LT/DOX NPs or PLT/DOX NPs (at an equivalent dose of 2.5 mg kg^{-1} DOX) every 2 days for a total of 4 times. At day 19, the mice were sacrificed, and the lungs were collected and imaged. Then, the lung tissues were subjected to H&E staining.

19. Statistical Analysis. Data were expressed as the means ± standard deviations (SD). Statistical analysis was performed by one-way ANOVA or Student's t-test. Significant differences between groups are indicated by $*p < 0.05$, $**p < 0.01$ and $***p < 0.001$.

Supplementary Tables

Table S1. Characterization of NPs conjugated by different amount of LMWH, TOS and PBA. (data represent mean data ± SD, n=3).

	LMWH:TOS	Average diameter (nm)	PDI	Zeta potential (mV)	CMC[2] (mg mL^{-1})
LT NPs	1:4	236.7±2.3	0.204±0.034	-31.2±4.3	5.91×10^{-2}
	1:2	218.5±2.1	0.226±0.021	-33.1±3.6	
	1:1	141.3±1.1	0.142±0.042	-30.1±3.4	
	1:1/2	198.42±3.2	0.352±0.033	-34.6±2.8	
	1:1/4	233.21±2.2	0.421±0.031	-35.2±4.1	
PLT NPs	1:1	143.1±2.6	0.121±0.033	-26.7±3.0	4.87×10^{-2}

Table S2. Characterization of different DOX-loaded NPs (data represent mean data ± SD, n=3).

	Conjugate : DOX	Size (nm)	PDI	DOX loading capacity(%)
LT/DOX NPs	9:1	140.7±1.3	0.162±0.021	9.1±1.0
	19:1	143.1±2.1	0.210±0.022	4.5±0.7
PLT/DOX NPs	9:1	142.8±3.3	0.188±0.017	8.8±1.1
	19:1	144.1±3.0	0.132±0.038	4.4±0.3

Table S3. Pharmacokinetic parameters of DOX after intravenous administration of free DOX, LT/DOX NPs, PLT/DOX NPs or PLT-Fru/DOX NPs at a dose of 3 mg kg^{-1} DOX.(data represent mean data ± SD, n=3)

	$t_{1/2\beta}$ (h)	V1(L Kg^{-1})	CL(L h^{-1} Kg^{-1})	AUC(0-t) (mg $L^{-1}h^{-1}$)	AUC(0-∞) (mg $L^{-1}h^{-1}$)
Free DOX	0.356±0.080	0.169±0.083	4.808±0.455	0.547±0.050	0.624±0.049
LT/DOX NPs	5.108±0.232	0.962±0.110	0.760±0.017	3.168±0.243	3.945±0.280
PLT/DOX NPs	4.619±0.344	0.719±0.062	0.766±0.011	3.278±0.141	3.917±0.256
PLT-Fru/DOX NPs	7.133±0.380	0.855±0.118	0.535±0.005	5.040±0.270	5.612±0.358

SUPPLEMENTARY FIGURES

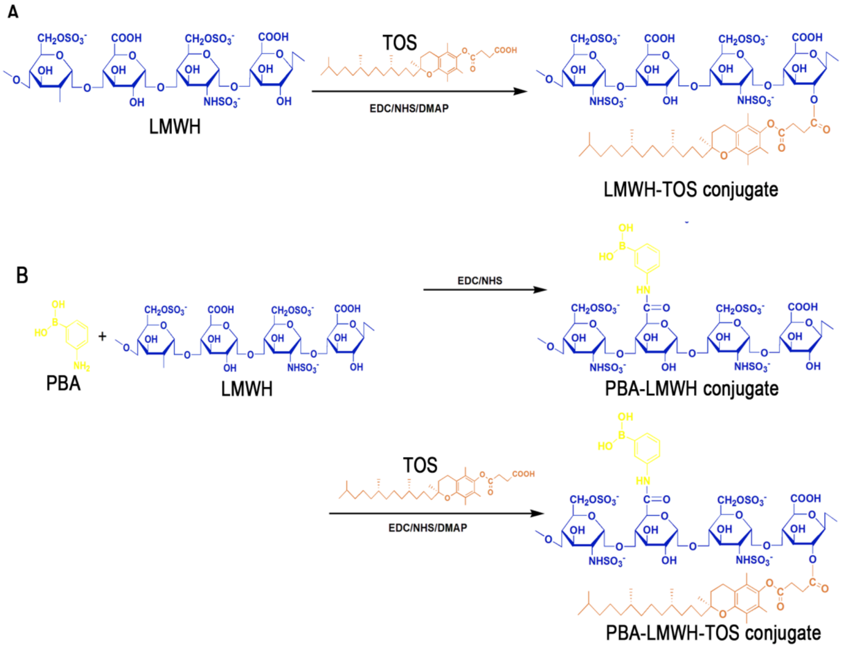

Figure S1. (A) The synthesis procedures and chemical structures of LMWH-TOS conjugate and (B) PBA-LMWH-TOS conjugate.

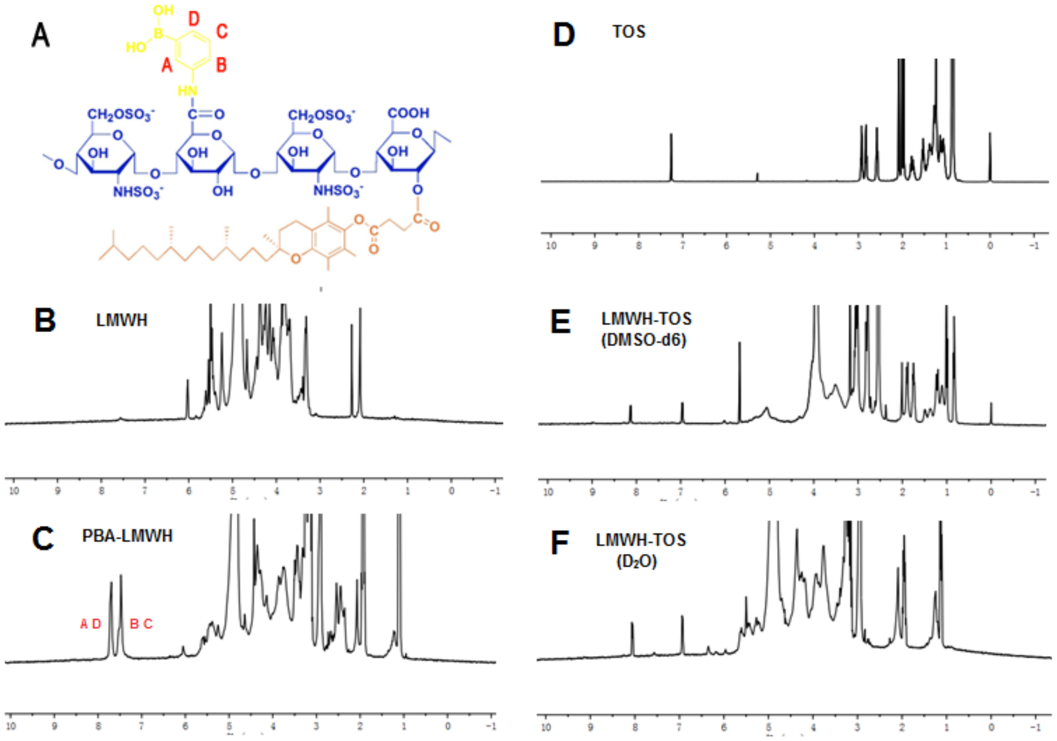

Figure S2. (A) Chemical structure of PBA-LMWH-TOS conjugate. The ^{1}H-NMR spectrums of (B) LMWH (in D_2O), (C) PBA-LMWH (in D_2O), (D) TOS (in $CDCl_3$), (E) LMWH-TOS (in DMSO-d6) and (F) LMWH-TOS (in D_2O).

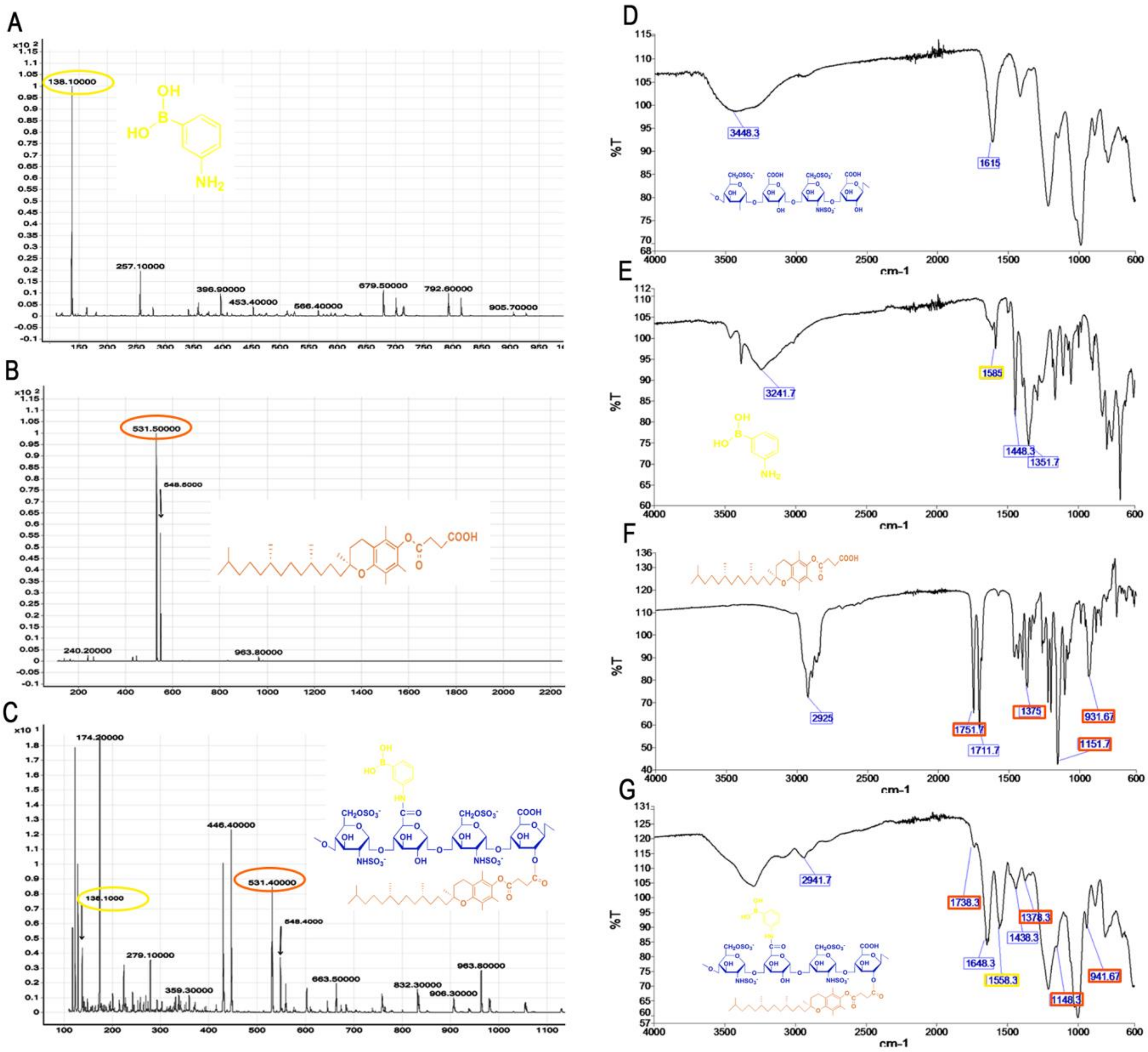

Figure S3. The mass spectra of PBA (A), TOS (B) and PBA-LMWH-TOS (C) conjugate. PBA: $[M+H]^+=138.1$; TOS: $[M+H]^+=531.5$, $[M+NH_4]^+=548.5$. The IR spectra of LMWH (D), PBA (E), TOS (F) and PBA-LMWH-TOS conjugate (G).

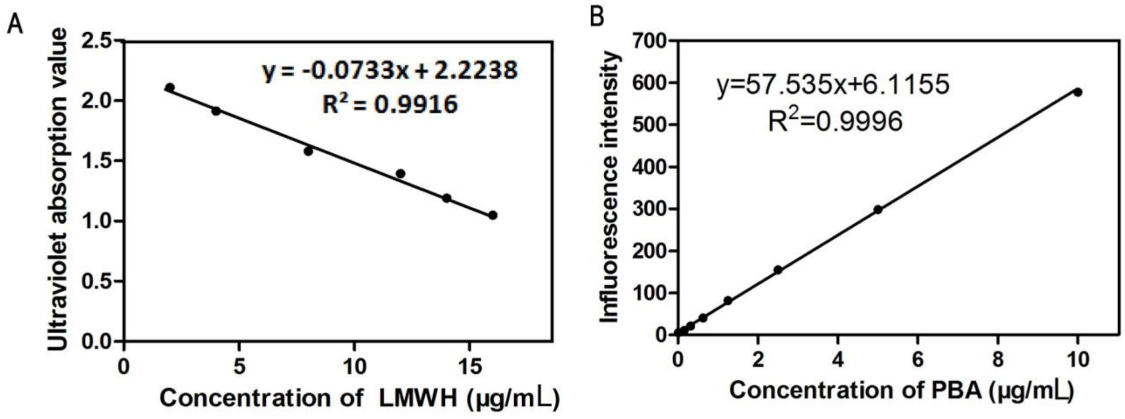

Figure S4. (A) The standard curve of LMWH determined by toluidine blue assay. (B) The fluorescence standard curve of PBA. (E_x = 302 nm and E_m = 375 nm).

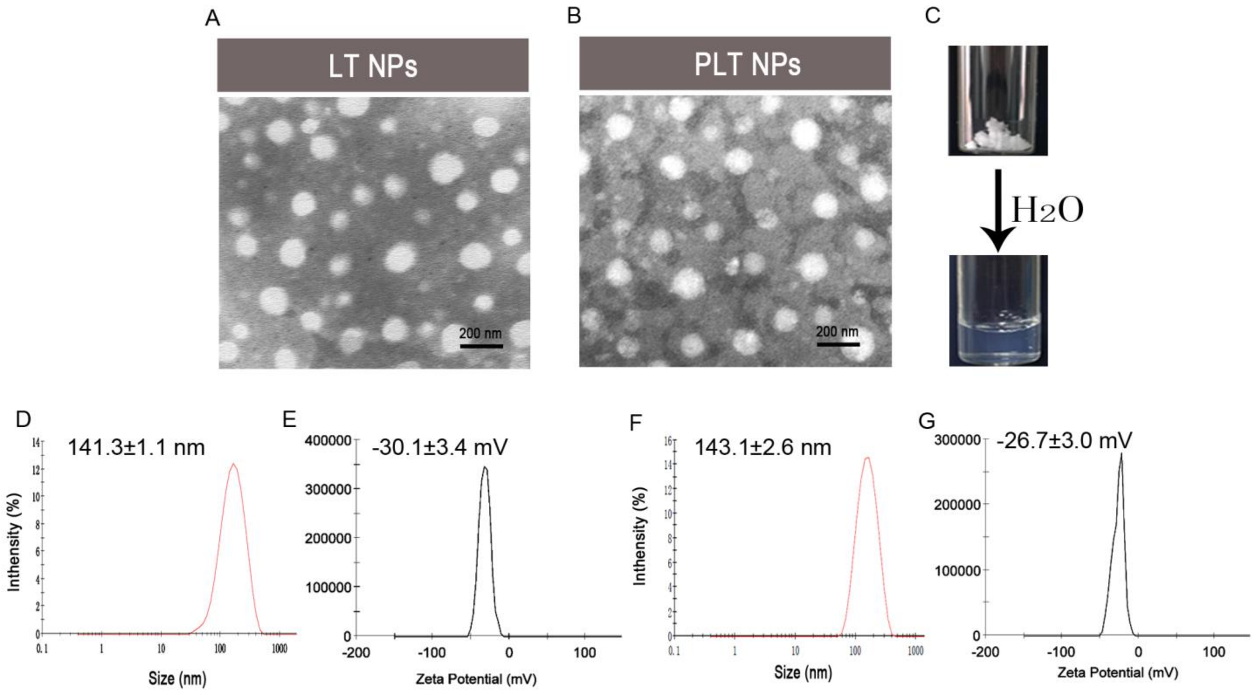

Figure S5. The transmission electron microscopy (TEM) image of LT NPs (A) and PLT NPs (B). (C) The powder of PBA-LMWH-TOS conjugate and the solution of PBA-LMWH-TOS micelles. The DLS of LT NPs (D) and PLT NPs (F).The Zeta potential of LT NPs (E) and PLT NPs (G).

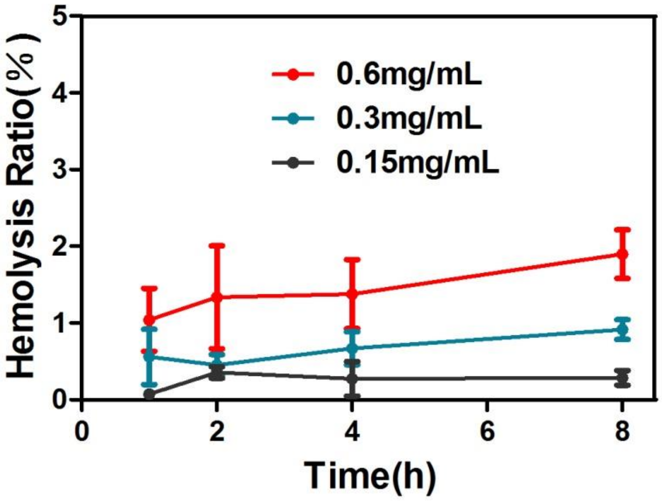

Figure S6. The hemolysis ratio (%) of red blood cell cultured with LT NPs at various concentrations within 8 h. Hemolysis ratio less than 5% was regarded as no obvious hemolysis (Means ± SD, n = 3).

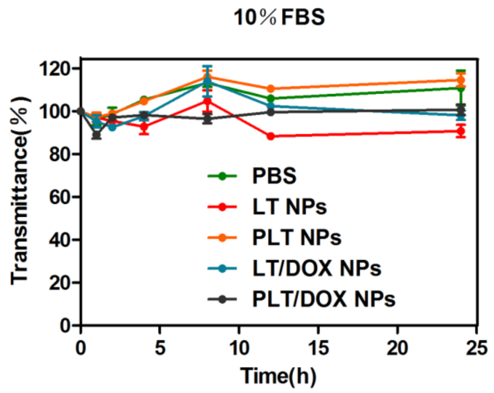

Figure S7. The serum stability of NPs within 24 h incubation with 10% FBS at 37 ℃, respectively (Means ± SD, n = 3).

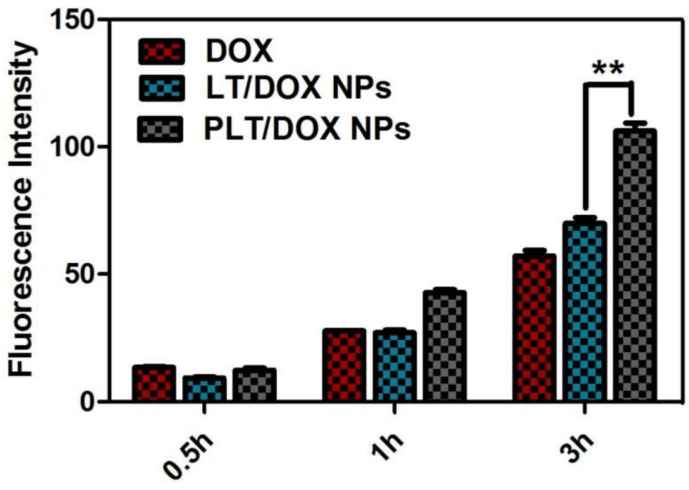

Figure S8. Quantitative cellular uptake of HepG2 cells after incubation with Free DOX, LT/DOX NPs and PLT/DOX NPs respectively for 0.5 h, 1 h and 3 h (Means ± SD, n = 3). ** indicates p < 0.01.

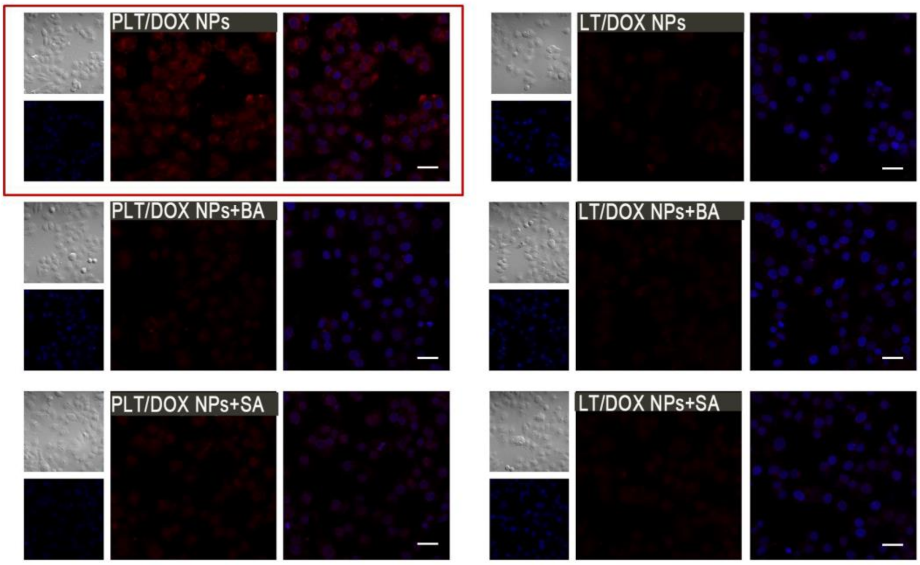

Figure S9. CLSM images of competitive inhibition study on HepG2 cells. Cells were treated with PLT/DOX NPs, PLT-BA/DOX NPs, PLT-SA/DOX NPs, LT/DOX NPs, LT-BA/DOX NPs and LT-SA/DOX NPs (at equivalent dose of DOX 10 μg/mL^{-1}, the concentration of free SA and PBA is 500 μM) respectively for 2 h, including DOXchannel (red) and DAPI-stained nucleus channel (blue). The scale bar indicates 100 μm.

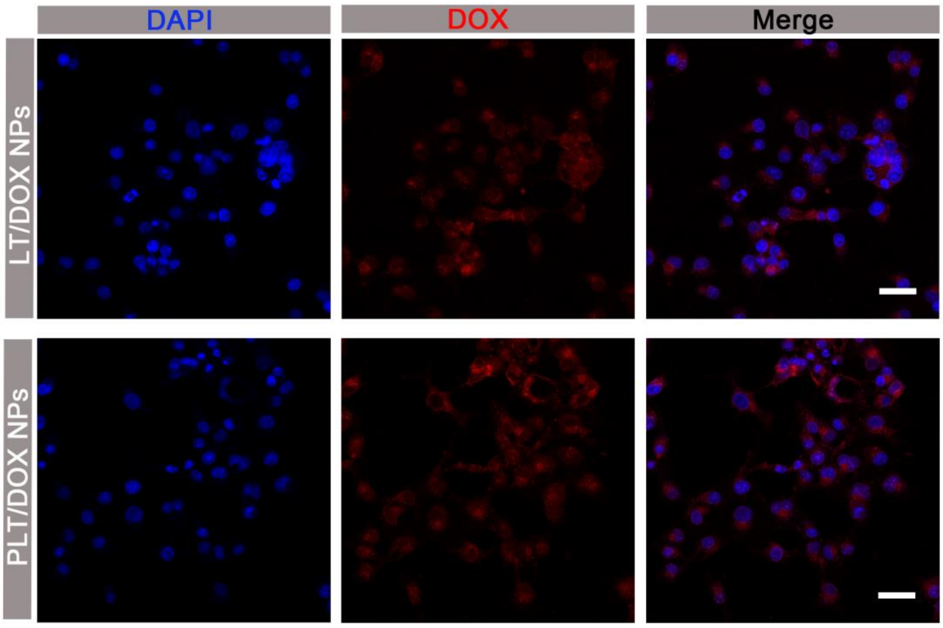

Figure S10. CLSM images for COS-7 cells after incubation with LT/DOX NPs and PLT/DOX NPs for 2 h respectively, including DOX channel (red) and DAPI-stained nucleus channel (blue). The scale bar indicates 100 μm.

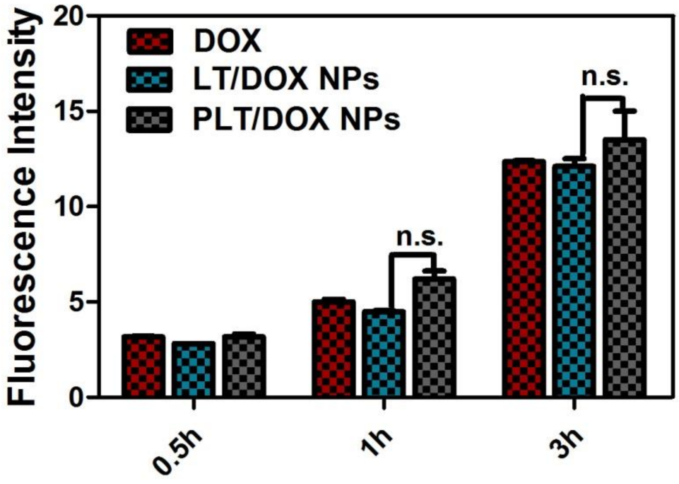

Figure S11. Quantitative cellular uptake of COS-7 cells after incubation with DOX, LT/DOX NPs and PLT/DOX NPs respectively for 0.5 h, 1 h and 3 h (Means ±SD, n = 3). n.s. indicates $p > 0.05$.

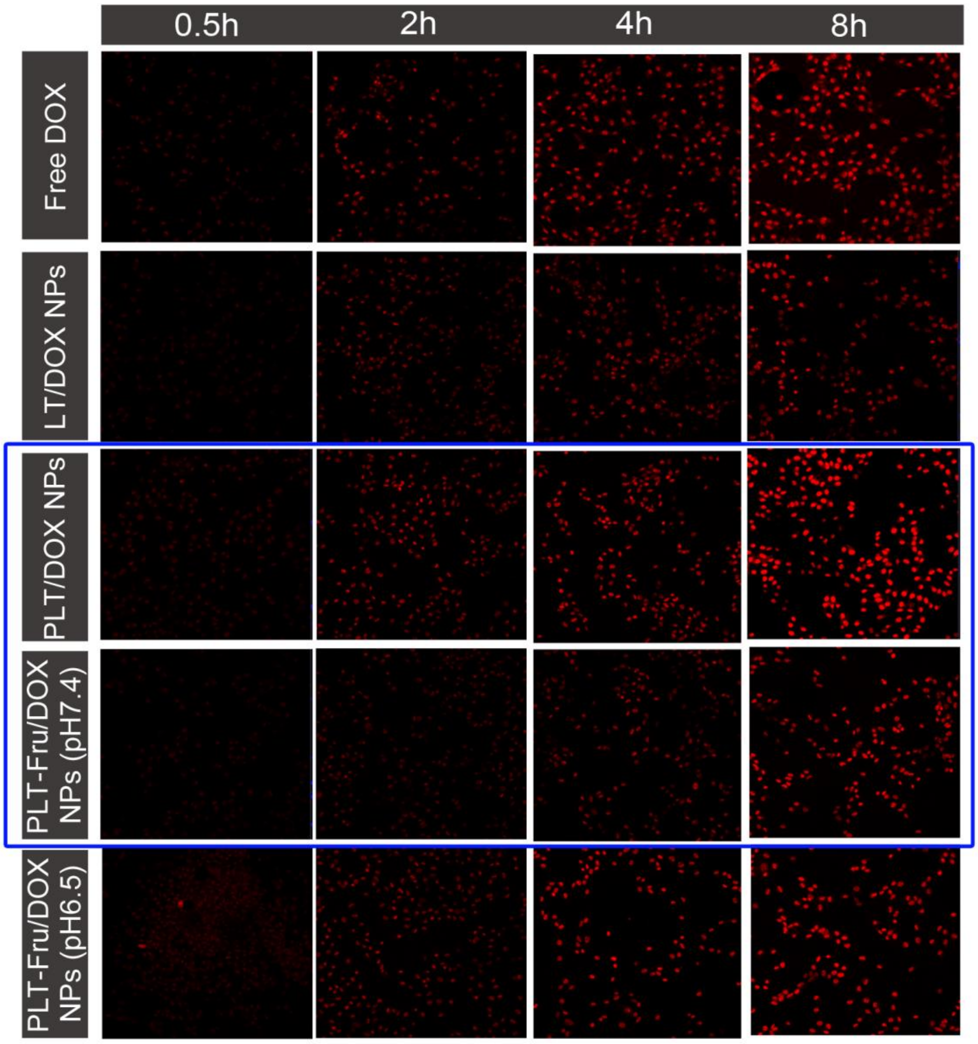

Figure S12. CLSM images of B16F10 cells after the incubation with free DOX, LT/DOX NPs, PLT/DOX NPs, PLT-Fru/DOX NPs (pH 7.4) or PLT-Fru/DOX NPs (pH 6.5) for 0.5 h, 2 h, 4 h and 8 h.

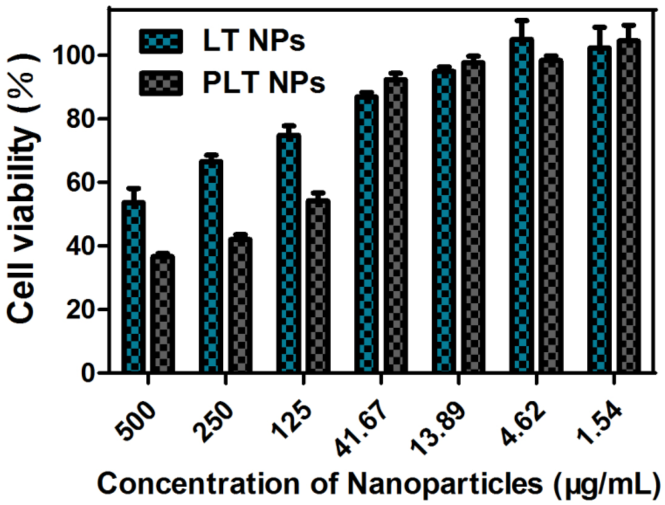

Figure S13. Cytotoxicity evaluation of LT NPs and PLT NPs using MTT assay (Means ± SD, n = 4).

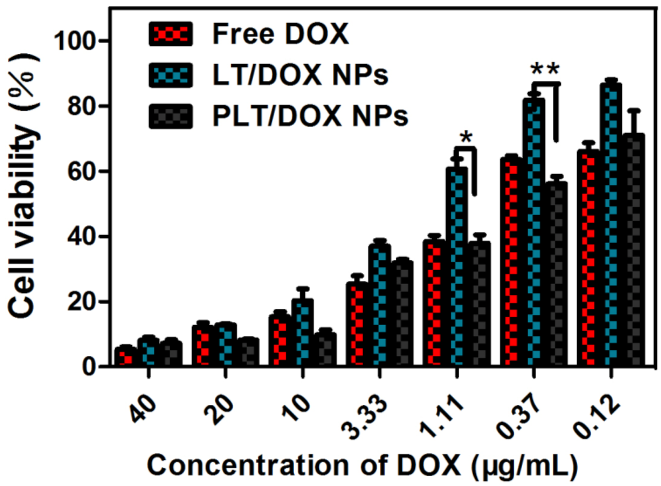

Figure S14. Cytotoxicity evaluation of Free DOX, LT/DOX NPs and PLT/DOX NPs using MTT assay. (Means ± SD, n = 4). * indicates $p < 0.05$ and ** indicates $p < 0.01$.

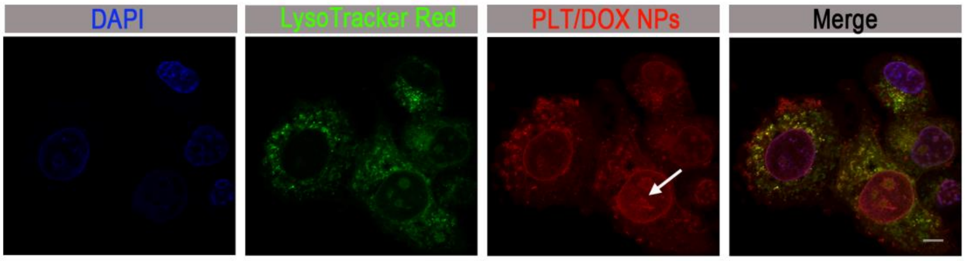

Figure S15. CLSM images of HepG2 cells after incubation with PLT/DOX NPs for 2 h, including Lysotracker Red-stained lysosome channel (green), DOX channel (red) and DAPI-stained nucleus channel (blue). The arrow indicated the signal of DOX in nucleus The scale bar indicates 10 μm.

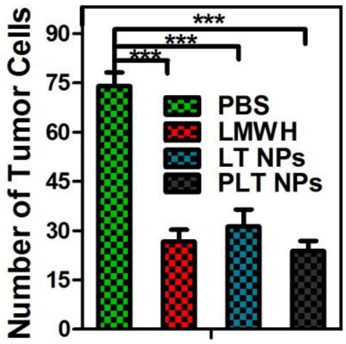

Figure S16. The number of implanted B16F10 tumor cells in lung sections, counting the tumor cell number randomly selected from five views (Means ±SD, n = 3). *** indicates $p < 0.001$.

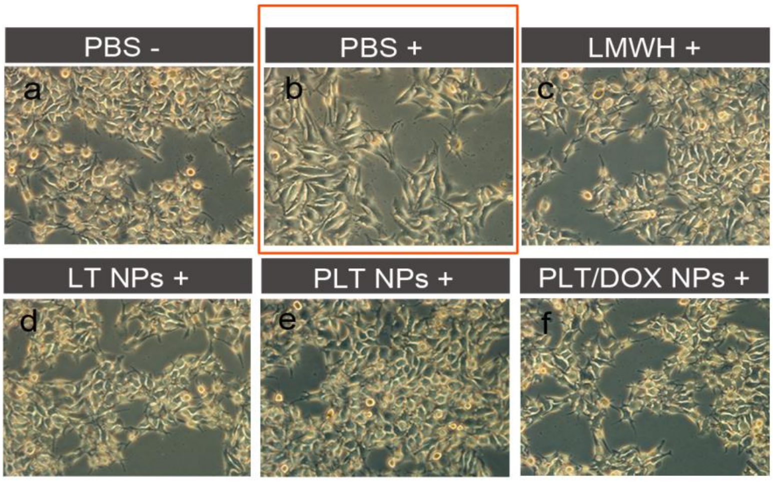

Figure S17. The images of the morphology of B16F10 cells after separate incubation with PBS-, PBS+, LMWH+, LT NPs+, PLT NPs+, and PLT/DOX NPs+ for 30 h . "+" means co-incubation with platelets after administration.

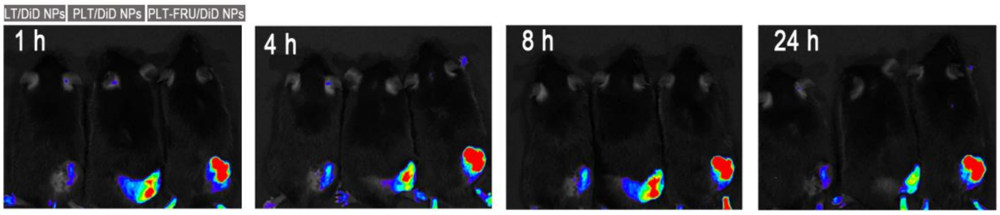

Figure S18. Representative living images of B16F10 tumor-bearing mice at 1 h, 4 h, 8 h and 24 h after systemic administration of LT/DiD NPs, PLT/DiD NPs or PLT-Fru/DiD NPs respectively.

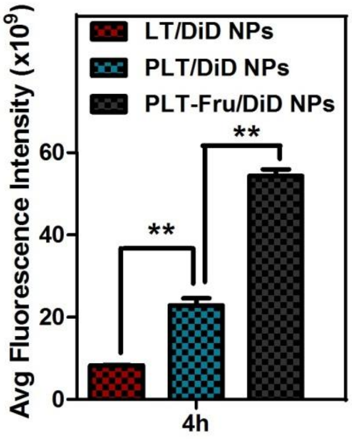

Figure S19. Average fluorescence intensity semi-quantitative results of B16F10 tumor-bearing mice at 4 h after the systemic administration of DiD-loaded NPs *in vivo*. Means ± SD, n = 3, ** indicates $p < 0.01$.

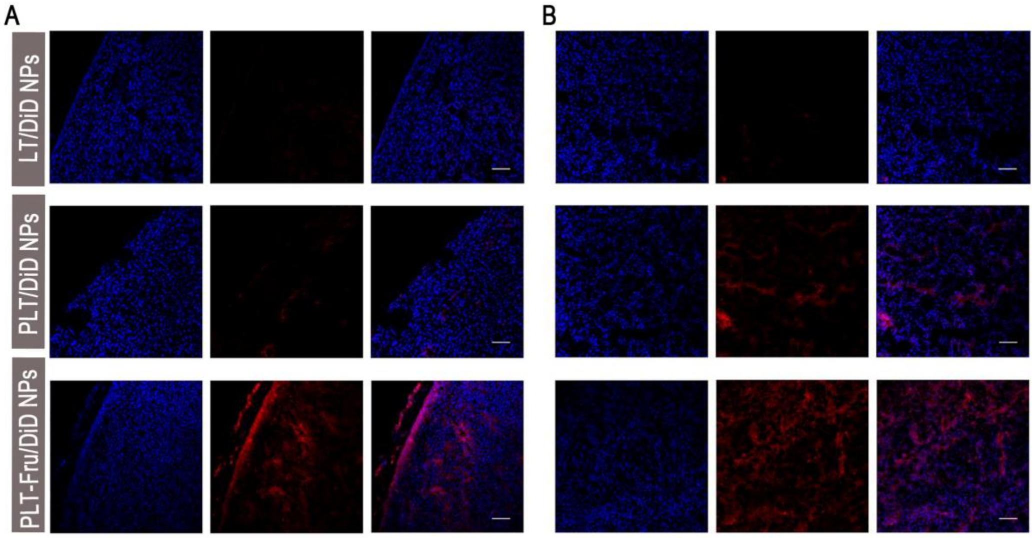

Figure S20. CLSM images of tumor tissue frozen sections from B16F10 tumor-bearing mice 24 h after systemic administration of DiD-loaded NPs (red). Cell nucleuses were stained with DAPI (blue). Image (A) showed the marginal area of tumors. Image (B) showed the central area of tumors. Scale bar indicates 200 μm.

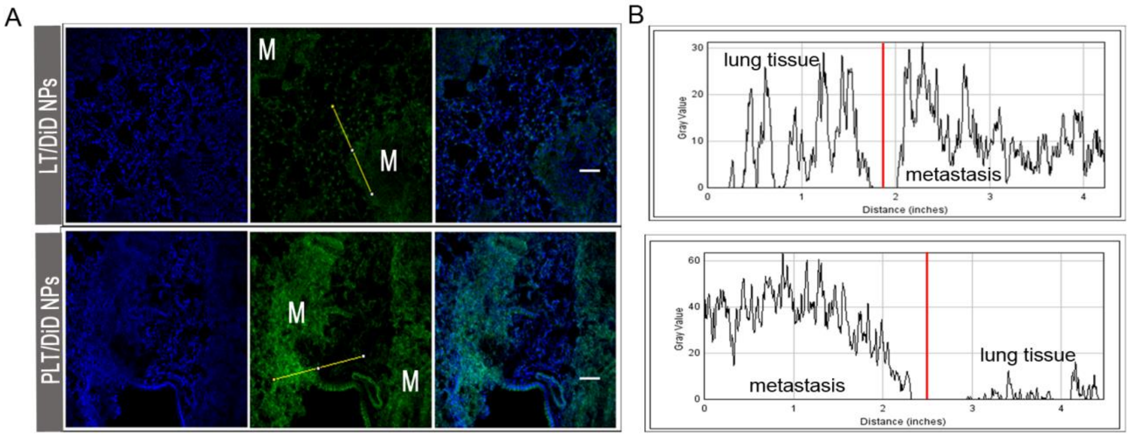

Figure S21. (A) Confocal images of lung sections from B16F10 metastasis model mice at 6 h after systemic administration of DiD-loaded NPs (green). Cell nuclei were stained with DAPI (blue). M indicates the metastases in lung. The scale bar represents 200 μm. (B) The signal intensity of DiD-loaded NPs in normal lung tissue and metastatic nodule calculated by Image J.

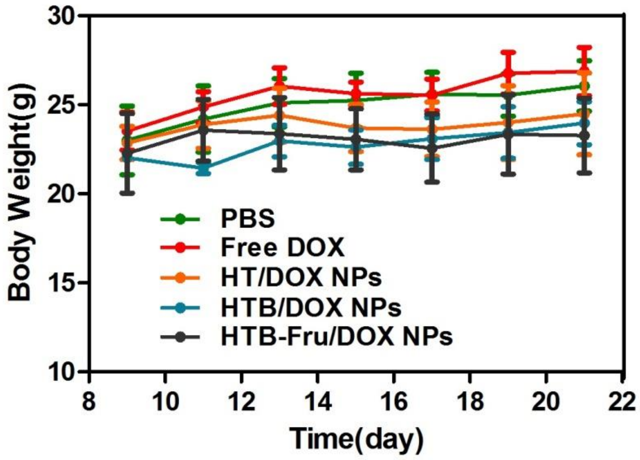

Figure S22. Body weight variation curves of the B16F10 tumor-bearing mice during the indicated therapy.

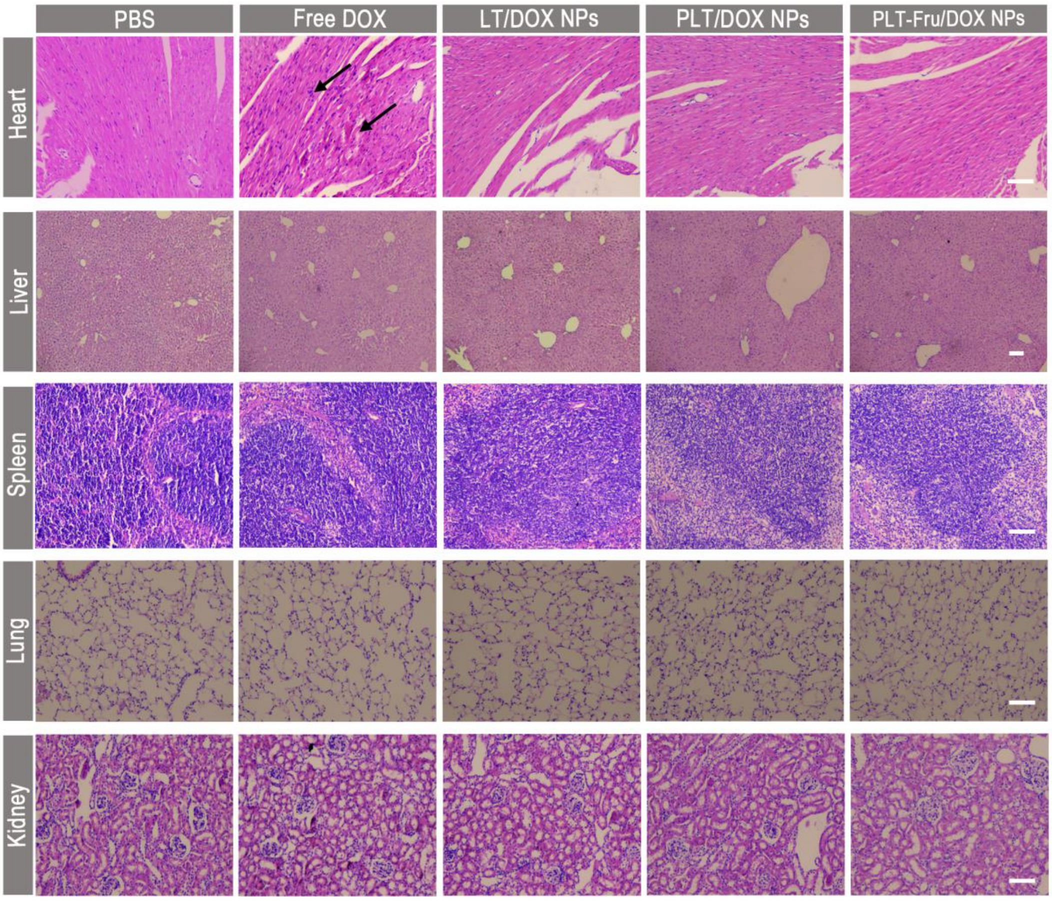

Figure S23. H&E staining assays of major organs collected from B16F10 tumor-bearing C57BL/6 mice on the 21th day after a total of 5 times administration with PBS, free DOX, LT/DOX NPs, PLT/DOX NPs and PLT-Fru/DOX NPs respectively. Black arrows indicate the location of neutrophils and atrophic myocardial cells. Scale bar indicates 100 μm.

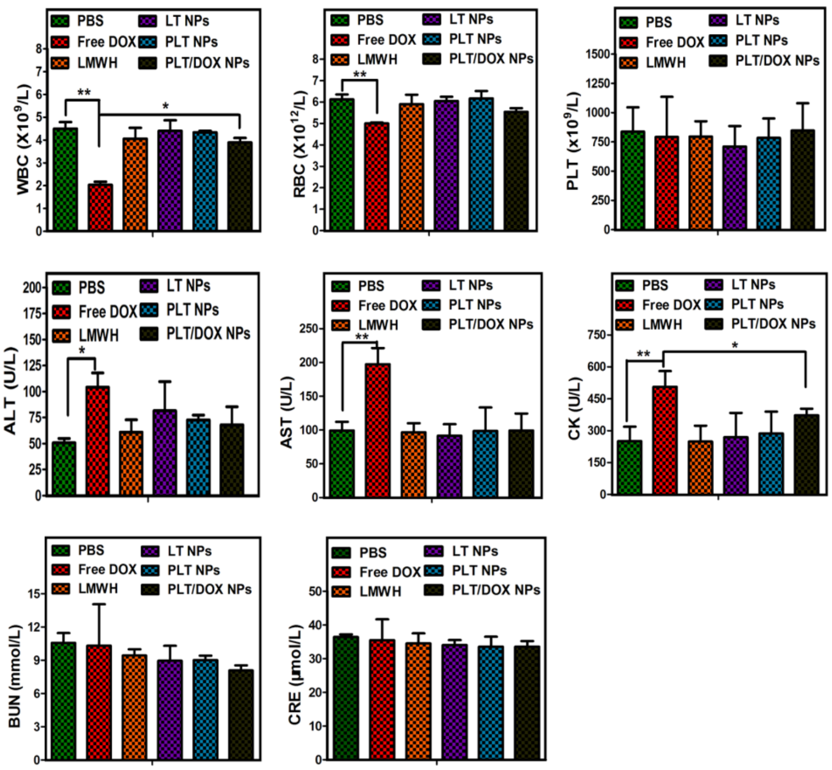

Figure S24. Blood routine examination and serum biochemistry data of mice treated with PBS, free DOX, LMWH, LT NPs, PLT NPs and PLT/DOX NPs (at an equivalent dose of 3 mg kg^{-1} DOX) respectively in a total of 5 times. (n =4, Means ± SD). * indicates $p < 0.05$, ** indicates $p < 0.01$.

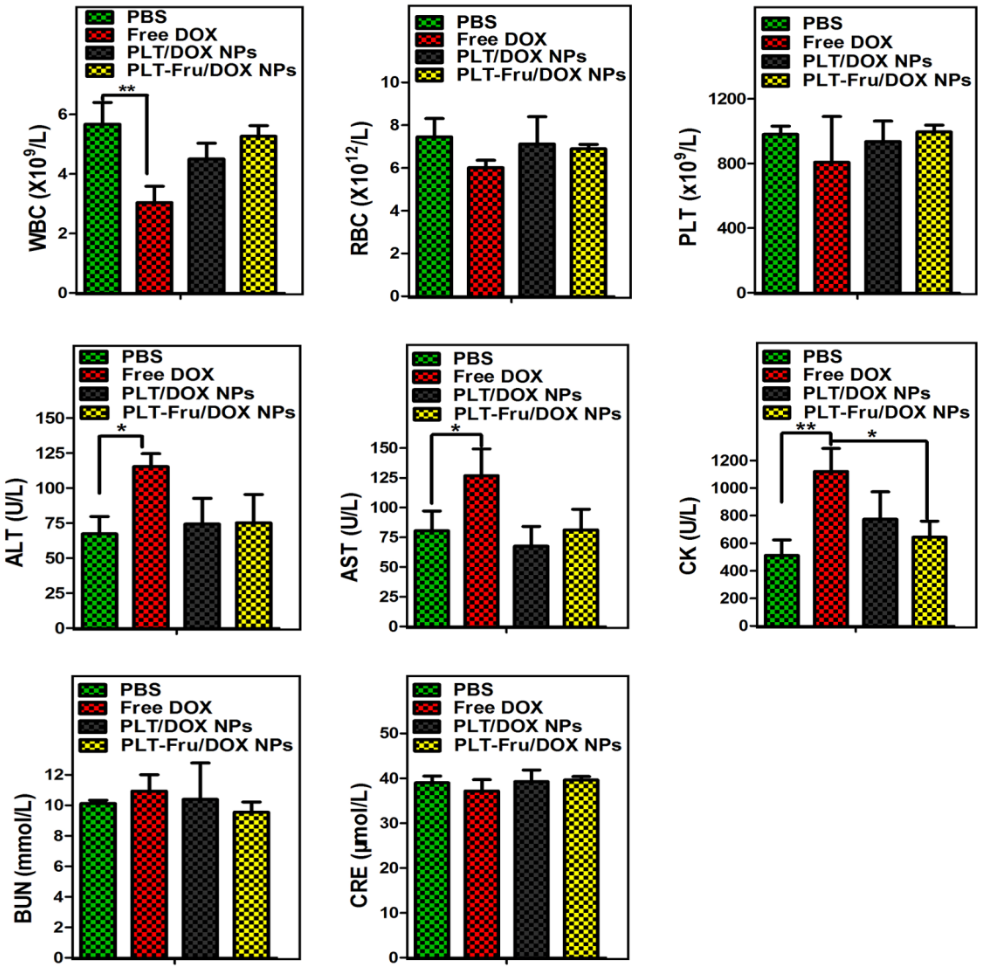

Figure S25. Preliminary safety evaluation of the "Fru-blocking" strategy. Blood routine examination and serum biochemistry data of mice treated with PBS, free DOX, PLT/DOX NPs and PLT-Fru/DOX NPs (at an equivalent dose of 3 mg kg^{-1} DOX) respectively in a total of 5 times. (n = 4, Means ± SD). * indicates $p < 0.05$, ** indicates $p < 0.01$.

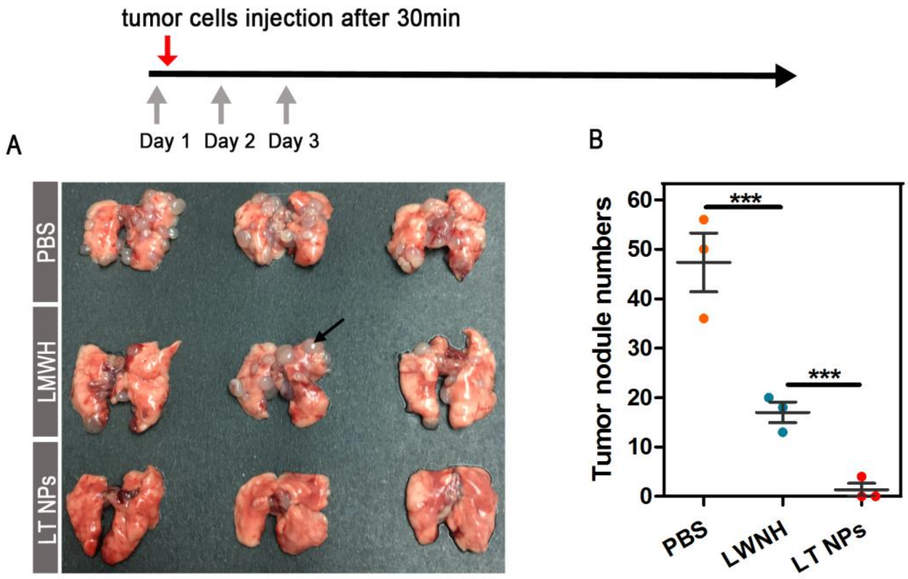

Figure S26. (A) Images of lungs harvested from the CT26 metastasis Balb/c mice model on the 20th day after treatment with PBS, free LMWH and LT NPs respectively by intravenous injection (at dose of 60 mg kg^{-1}). The black arrow indicated the metastatic nodule (B) The number of CT26 metastatic nodules on the surface of lungs (Means ± SD, n = 3). *** indicates $p < 0.001$.

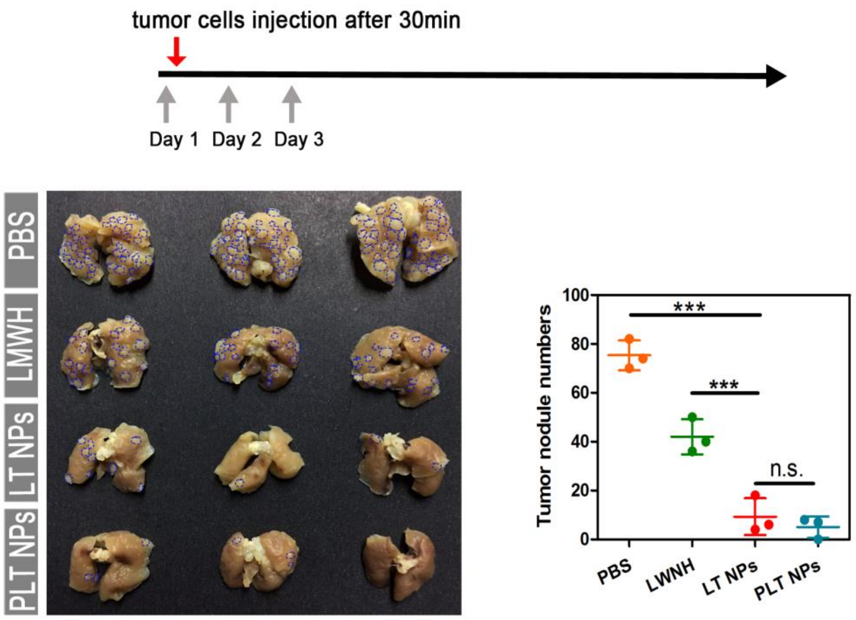

Figure S27. (A) Images of lungs harvested from the 4T1 metastasis Balb/c mice model on the 20th day after treatment with PBS, free LMWH, blank LT NPs and blank PLT NPs respectively by intravenous injection (at dose of 60 mg kg−1). (B) The number of 4T1 metastasis nodules on the surface of lungs (means ± SD, n = 3). *** indicates $p < 0.001$.

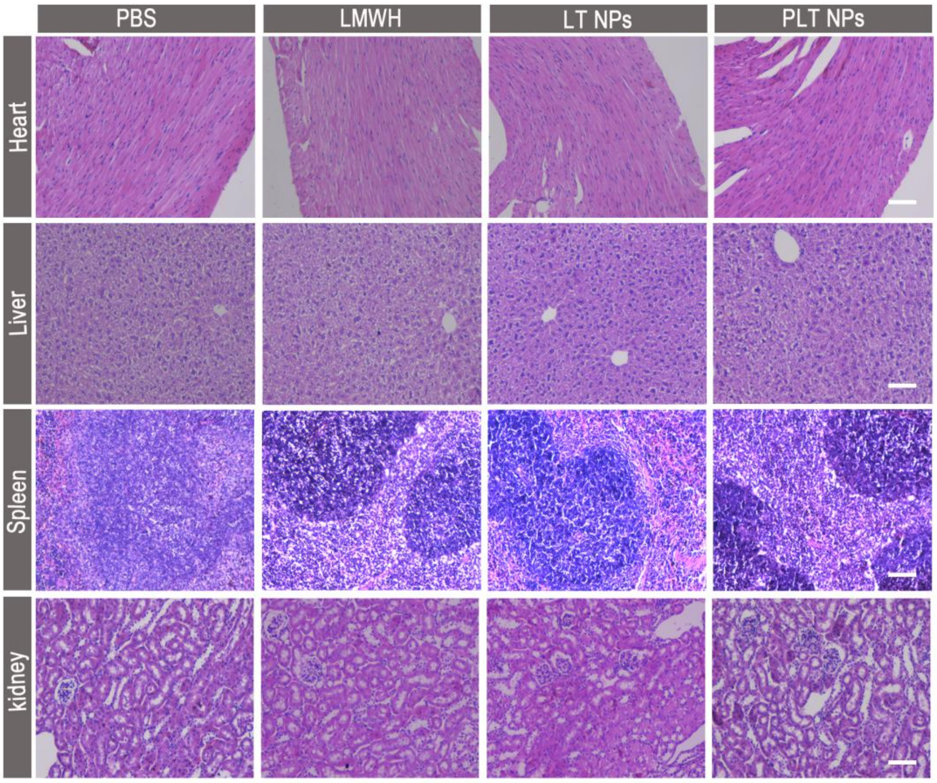

Figure S28. H&E staining assays of major organs collected from B16F10 metastasis C57BL/6 mice model on the 20th day after administration with PBS, LMWH, blank LT NPs and PLT NPs respectively in anti-metastasis assay on B16F10 metastastis mouse model *in vivo*. Scale bar indicates 100 μm. No obvious damage of tissues was observed.

References

[1] T. Zong, L. Mei, H. Gao, K. Shi, J. Chen, Y. Wang, Q. Zhang, Y. Yang, Q. He. *J. Pharm. Sci.* **2014**, 103, 3891−3901.

[2] L. Mei, Y. Liu, C. Xia, Y. Zhou, Z. Zhang, Q. He, *Mol. Pharmaceutics* **2017**, *14*, 513.
